# Chasing bacterial *chassis* for metabolic engineering: a perspective review from classical to non‐traditional microorganisms

**DOI:** 10.1111/1751-7915.13292

**Published:** 2018-06-21

**Authors:** Patricia Calero, Pablo I. Nikel

**Affiliations:** ^1^ The Novo Nordisk Foundation Center for Biosustainability Technical University of Denmark 2800 Kongens Lyngby Denmark

## Abstract

The last few years have witnessed an unprecedented increase in the number of novel bacterial species that hold potential to be used for metabolic engineering. Historically, however, only a handful of bacteria have attained the acceptance and widespread use that are needed to fulfil the needs of industrial bioproduction – and only for the synthesis of very few, structurally simple compounds. One of the reasons for this unfortunate circumstance has been the dearth of tools for targeted genome engineering of bacterial *chassis*, and, nowadays, synthetic biology is significantly helping to bridge such knowledge gap. Against this background, in this review, we discuss the state of the art in the rational design and construction of robust bacterial *chassis* for metabolic engineering, presenting key examples of bacterial species that have secured a place in industrial bioproduction. The emergence of novel bacterial *chassis* is also considered at the light of the unique properties of their physiology and metabolism, and the practical applications in which they are expected to outperform other microbial platforms. Emerging opportunities, essential strategies to enable successful development of industrial phenotypes, and major challenges in the field of bacterial *chassis* development are also discussed, outlining the solutions that contemporary synthetic biology‐guided metabolic engineering offers to tackle these issues.

## Introduction

Decades of research considerably expanded the repertoire of biological functions that microbial cells can incorporate into their physiological and metabolic agendas. Nowadays, *designer cells* can be constructed by adopting a combination of genome editing tools, chemical DNA synthesis and DNA assembly technologies – thereby fulfilling the practical goal of synthetic biology, that is, the construction of living cells from individual parts, which are purposefully assembled to yield a functional entity (Jullesson *et al*., 2015). Some important hurdles, however, have been just started to be taken into consideration. Cells naturally control the gene expression flow using a sophisticated variety of RNA, protein and DNA‐modifying layers of regulation, and these regulatory networks enable them to effectively respond to their environments and external cues. Therefore, adding novel, genetically encoded biological functions to these complex networks is expected to have different consequences depending on the host in which they are introduced into, as these regulatory layers would be likewise different across species. Several bacterial hosts have been adopted for plugging‐in and plugging‐out genetic circuits for specific purposes and, in most cases, the selection of the host cell was merely dictated by its availability and/or by historical tradition. However, synthetic biology ultimately aims at programming cells that can execute the implanted functions in a predictable fashion, and such objective thus calls for the adoption of specific, *formatted* hosts that will necessarily have different properties depending on the application envisioned. In the broadest sense of the term, a biological *chassis* can be defined as the physical, metabolic and regulatory containment for plugging‐in and plugging‐out dedicated genetic circuits and regulatory devices. This wide definition incorporates a clear engineering standpoint, in which a set of predefined parts are assembled together in a rational, standardized way leading to the final object (Endy, 2005). Moreover, and as proposed by Danchin and Sekowska (2013), the concept of *chassis* encompasses the notion that there is a clear distinction between a program or *software* that encodes the target function(s), and a machine or *hardware* (i.e. the *chassis* itself) that expresses and executes the program.

Apart from their role in the context of synthetic biology, the adoption of suitable bacterial *chassis* has a considerable impact in the field of metabolic engineering (Stephanopoulos, 2012; Nielsen and Keasling, 2016; Smanski *et al*., 2016). The integration of synthetic biology tools and strategies into advanced metabolic engineering has enabled the incorporation of a number of non‐traditional microorganisms as hosts for developing efficient microbial cell factories. The (already extensive) list of the microbial hosts that can be adopted for such purposes continues to expand as more tools for precise gene and genome manipulation become available. Yet, the implementation of a given host for practical applications seems to be still governed by some degree of randomness, and a remarkably small number of microbial cell factories have achieved full commercial exploitation. This situation contrasts with the unparalleled momentum that the development of industrial microbial processes is gaining nowadays, driven by increased concerns about environmental issues and the prospect of dwindling petroleum resources worldwide that has increasingly shifted the industrial focus towards the use of microorganisms as biocatalysts. Yet again, the production of only a limited number of compounds has successfully reached commercial scale, which indicates how difficult the whole process of bringing a product into the market is. Improving yields, titres and productivity of microbial processes to enable commercialization requires rational manipulation of the microbial physiology, stress responses, and, even more importantly, engineering the core metabolism in the selected *chassis*. An often overlooked aspect is that the *chassis* has to be adapted to specific substrate(s) and its impurities in an industrial setup, which ultimately dictates the needs and characteristics of downstream processing. Consequently, a major topic in the field of metabolic engineering is the selection of an optimal *chassis* not only able to execute the functions needed for efficient bioproduction, but also hefty enough to tolerate the harsh operating conditions characteristic of industrial processes, which are of course different from the mild, controlled conditions that prevail in the laboratory.

In general, the physical and spatial shape of a microbial cell, its genomic complement, its default gene expression machinery and the complement of metabolic functions are not enough to automatically meet the requirements of a suitable *chassis*, and further manipulations are needed in order to fulfil the characteristics desirable for specific applications. Building on the wealth of information generated over the years, in this review we discuss the state of the art in bacterial *chassis* development, presenting the main advantages and limitations of traditional hosts (including a summary of recent efforts on development of dedicated tools), also bringing under the spotlight novel bacterial species that hold promise for future developments. For the sake of the present discussion, we will restrict the scope of this review to bacterial *chassis*, without forgetting that the development of other microbial platforms (e.g. yeast and filamentous fungi) continues to gain considerable attention for both fundamental and practical applications. Finally, we also present and discuss what we perceive as being the main challenges that need to be overcome in the design and construction of bacterial *chassis* in order to reach a level of maturity compatible with commercial exploitation.

## Desirable properties in the ideal bacterial *chassis*: bridging the knowledge‐to‐application gap through synthetic biology

**Figure 1:**
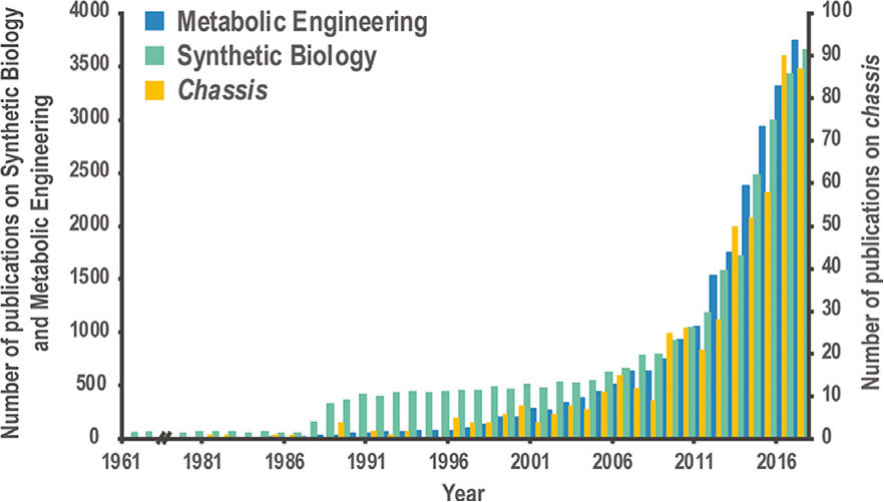
Intersection between the adoption of microbial *chassis* and the fields of metabolic engineering and synthetic biology, as reflected in the relevant literature since 1961 up to date. The diagram indicates the number of times that the words ‘Metabolic Engineering’ (*blue*), ‘Synthetic Biology’ (*green*) and ‘*chassis*’ (*yellow*) have been used as keywords in research and review articles in the field literature over the years (source: PubMed, accessed in May 2018). Note that the scale is different for ‘*chassis*’ (indicated to the right of the diagram) and both ‘Metabolic Engineering’ and ‘Synthetic Biology’ (indicated to the left of the diagram).

Over the past two decades, and together with the explosive expansion of the fields of metabolic engineering and synthetic biology, the development of suitable *chassis* that could host newly‐designed biological functions and processes has achieved substantial relevance, as it becomes apparent by inspecting the exponential increase in the number of publications on the topic over the last few years (Fig. 1). Only during the last year, almost 100 articles had the word ‘*chassis*’ listed as a keyword – a citation trend that mirrors the general increase in the number of publications on synthetic biology and metabolic engineering. This observation indicates how intimate the connection between new engineering endeavours and the adoption of specific hosts is. Yet, what would be the starting point in the process of selection and development of a bacterial *chassis* for this purpose?

### From ‘built‐in’ properties to emergent bacterial phenotypes

Ideally, a microbial *chassis* should possess certain features to meet the practical necessities of metabolic engineers and to ensure an easy and fast construction workflow of reliable production strains. These features include (i) enough basic knowledge on the microorganism, setting the basis for the design of culture media and bioprocesses, (ii) simple nutritional requirements, including readily accessible carbon and nitrogen sources, (iii) ‘built‐in’ high resistance to physicochemical stress, (iii) fast and efficient growth, (iv) availability of (and possibility of developing novel) tools for targeted genome manipulations, (v) efficient secretion systems (either natural or amenable for engineering) to facilitate downstream purification steps, especially when heterologous protein production is the target, and (vi) tolerance to extreme conditions, for example high temperature. Needless to say, there are very few microorganisms that would naturally fulfil all these six traits (Beites and Mendes, 2015). Traditionally, only well‐characterized bacteria that have been studied over the years in a laboratory setup have been adopted as *chassis* for metabolic engineering. This is also the case of microorganisms with a long tradition in the fields of microbiology and biotechnology that have managed to reach an industrial scale of production. The (somewhat short) list of bacterial hosts falling into this category includes *Escherichia coli* (Pontrelli *et al*., 2018), *Bacillus subtilis* (Gu *et al*., 2018), *Streptomyces* sp. (Spasic *et al*., 2018), *Pseudomonas putida* (Nikel and de Lorenzo, 2018), and *Corynebacterium glutamicum* (Wendisch *et al*., 2016), for which extensive background fundamental knowledge has been amassed. The wide use of these well‐established *chassis* notwithstanding, there has been a renewed interest for bringing up‐and‐coming host alternatives to the metabolic engineering community, thus broadening the repertoire of *chassis* available. Nevertheless, establishing a new *chassis* encompasses challenges that need to be tackled and for which we propose a streamlined process as disclosed below.

**Figure 2:**
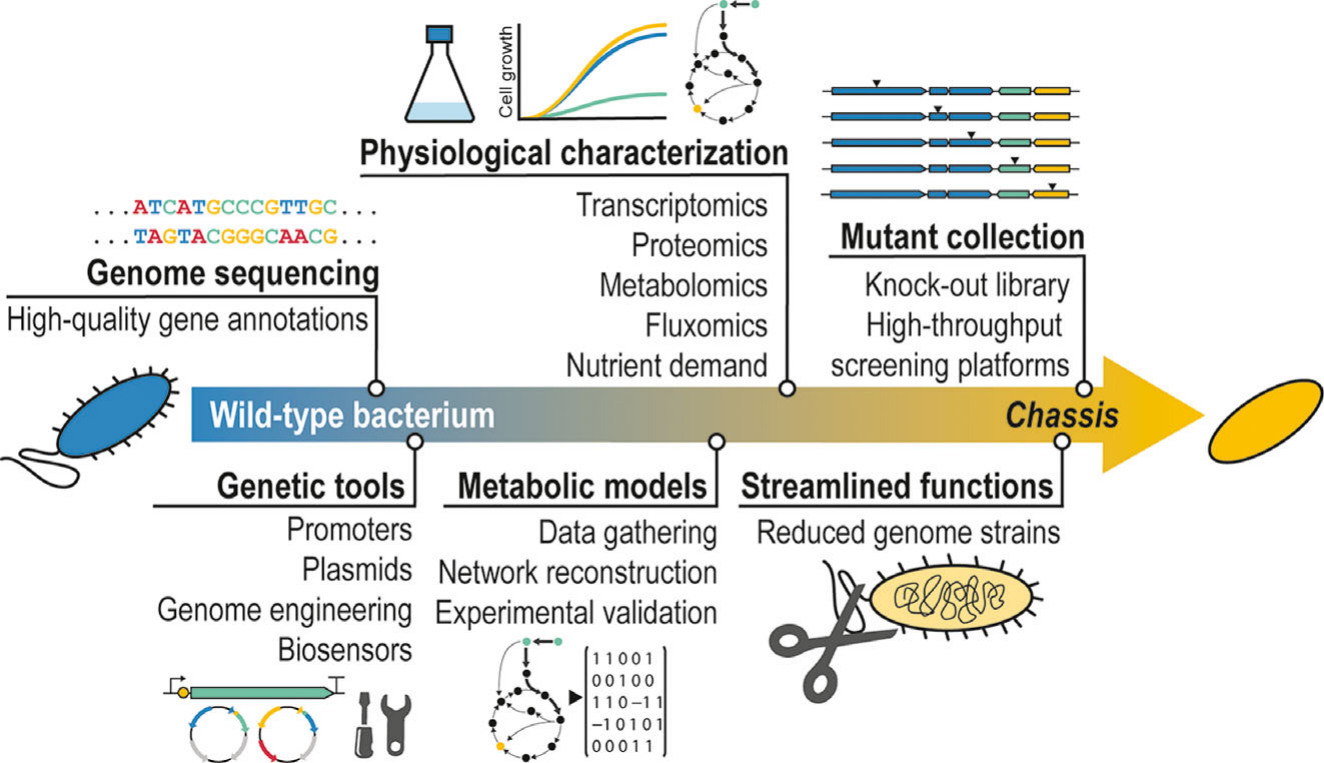
Proposed chart for the development of a bacterial *chassis* for metabolic engineering, indicating the key steps required for domestication of a potentially interesting wild‐type strain. The entire process builds upon six main interconnected aspects, which cover the whole range between gaining fundamental insight into functional genomics and physiology of the strain at stake and the design and adoption of dedicated synthetic biology tools. Although there is a structure to be followed along the process (that usually starts with the sequencing and expert annotation of the genome and a thorough physiological characterization), the steps indicated in the chart are not necessarily sequential in nature.

The very starting point in this process is frequently a not‐so‐well‐known microorganism (most usually, a natural microbial isolate), which might possess some of the traits of interest for the field (e.g. pre‐endowed high tolerance to physicochemical stress and sufficiently rapid growth on a simple culture medium). As recently proposed for the development of the so‐called *model organisms* for biotechnology (Liu and Deutschbauer, 2018), a given wild‐type bacterial strain proposed as a potential *chassis* has to be subjected to extensive studies to fully exploit its potential before its wide adoption becomes possible. The necessary steps to be taken into this direction are summarized in Fig. 2, and they include key approaches such as (i) detailed sequencing of the genome, followed by expert, well‐curated annotation of genes to evaluate *in silico* the metabolic potential (and potentially negative traits, as pathogenicity) of the host, (ii) development of genetic tools, including both replicative and suicide plasmids, characterized promoters covering a wide range of gene expression levels, and genome engineering tools, for example CRISPR/Cas9 devices, for precise genetic manipulations, (iii) experimental enrichment of the knowledge on metabolism and physiology, through the implementation of *omics* technologies and genome‐wide metabolic reconstructions (this is a crucial aspect of the whole process, as feeding *in silico* metabolic models with actual experimental data provides the basis for further refinement of the predictions), (iv) construction and testing of libraries of knock‐out mutants in non‐essential genes, and (v) continuous curation and updating of all the data gathered into publically available databases to reach the whole scientific community.

### In the quest of reduced‐ and minimal‐genome bacterial chassis by trimming out unnecessary functions

Minimal interference of the endogenous biochemistry with any given heterologous pathway plugged‐in into the metabolism of a target host is a desirable trait for metabolic engineering (de Lorenzo *et al*., 2015). However, this is not often the case as metabolic intermediates or final products of the pathway may (i) cause toxicity issues by themselves, (ii) become targets of non‐predicted enzymatic reactions (resulting in misrouting or, again, toxicity), and/or (iii) act as inhibitors of other functions. In all these possible scenarios, the consequence is a decrease in the metabolic efficiency of the target pathway(s) that could severely affect final titres and yields. The construction of cell factories based on a reduced or, ideally, minimal‐genome *chassis* has been proposed as a potential solution to avoid these issues (Martínez‐García and de Lorenzo, 2016). The premise here is that such *minimal cell factories* are designed for *specific* production purposes, containing the minimal information and functions needed to grow and execute heterologous pathway(s) for efficient production with little to no interference arising from native functions. The behaviour of these minimal cells factories is expected to be more predictable due to their decreased complexity, and they should also have a higher catalytic efficiency as they do not waste energy in transcribing and translating unnecessary genes that would otherwise give rise to likewise unnecessary functions (Choe *et al*., 2016). A major challenge here is cataloguing such ‘unnecessary’ genes and functions, which would strictly depend on environmental (e.g. industrial operation) conditions. From a fundamental perspective, the quest for the elusive minimal genome has been proposed as the way forward to get access to all necessary components defining a living cell – and then using this information for the construction of efficient biocatalysts. One possibility to tackle this challenge would be to start off by trying to identify all the functions that are ubiquitously present in extant bacterial genomes. This approach, however, takes for granted that genomic analysis offers access to ubiquitous cell structures (or, rather, gene sequences, which are seldom sufficient to predict structures) and not functions. A more heuristic approach to overcome this problem is the identification of *persistent genes*, that is, genes that tend to be present in a *quorum* of genomes with a preset conservation percentage threshold (Acevedo‐Rocha *et al*., 2013). This classification divides the (bacterial) genome into two components, the *paleome*, which encodes all the functions needed to reproduce the cell in its progeny, while replicating its genome, and the *cenome*, which allows the cell to belong to a specific environmental niche. Such classification would in principle enable the user to decide on the set of (dispensable) cell functions that can be erased in any given bacterial *chassis* for specific applications. Transcriptional data is also a very important information to be exploited in the selection of dispensable cell functions, as the expression of many genes (including many of those making up for the paleome) is, as indicated above, highly dependent on the specific culture conditions (Kim *et al*., 2013).

As mentioned above, it is important to stress out that the set of genes necessary to sustain life is not the same across different environmental conditions, and it will vary significantly depending on whether the cells are growing in a rich or a simple, minimal medium and/or if there are toxic elements and compounds present. Consequently, the construction of a minimal bacterial genome might not lead to robust growth across all conditions. Pathways that are crucial for optimal growth under specific stress conditions or environmental fluctuations, for instance, may not be deemed essential under homoeostatic, balanced growth conditions (Nogales *et al*., 2012). This aspect is especially relevant when these cells are meant to be used for bioproduction, as each target product may require special operating conditions. As a more general principle, a reduced set of genes should not be taken as a synonym of robustness or fast growth, as shown for bacteria *naturally* endowed with very small genomes, for example *Mycoplasma pneumoniae* (Yus *et al*., 2009).

Top‐down approaches have been traditionally employed in engineering endeavours for obtaining minimal‐genome bacterial *chassis*, in which the genome of wild‐type laboratory strains has been reduced as much as possible without impairing growth (at least under certain growth conditions). Not surprisingly, *E. coli* has been the main subject of dedicated genome reduction projects (Hashimoto *et al*., 2005; Pósfai *et al*., 2006; Hirokawa *et al*., 2013; Park *et al*., 2014), as well as other bacterial strains of industrial relevance, for example *B. subtilis* (Ara *et al*., 2007; Morimoto *et al*., 2008), *Streptomyces avermitilis* (Komatsu *et al*., 2010) and *Pseudomonas putida* (Leprince *et al*., 2012; Martínez‐García *et al*., 2014c; Lieder *et al*., 2015). Further information about other reduced‐genome bacterial strains constructed *via* top‐down approaches can be found in recent reviews (Xavier *et al*., 2014; Choe *et al*., 2016). Other strategies have been recently adopted for the construction of minimal‐genome bacterial cells. The significant advancement in fast and cost‐effective techniques for DNA synthesis, assembly and efficient sequencing has contributed to the *de novo* construction of synthetic *Mycoplasma mycoides* cells as part of the *Minimal Genome Project* running at the J. Craig Venter Institute (Hutchison *et al*., 2016). In this case, the authors have adopted a bottom‐up genome minimization approach (Gibson *et al*., 2010) that, apart from enabling the construction of such a minimal‐genome bacterial *chassis*, has shed light on the complexity of bacterial genome structures. The first proof‐of‐principle of the project consisted of a *de novo* synthesized, modified version of the 1,000,000‐bp long genome of *M*. *mycoides* that was implanted into a DNA‐free *Mycoplasma capricolum* ‘envelope’, resulting in strain *JCVI‐syn1.0*. Building on this first version of the minimal bacterial cell, an even smaller genome was synthesized (*JCVI‐syn3.0*), spanning 531,560 bp and 473 genes. A comparison of these two synthetic genomes revealed a common set of 256 genes, which probably represent the *authentic* minimal set of genes needed for (limited) cell viability. Surprisingly, almost one‐third of these genes in this synthetic construct encode unknown functions. Recent efforts by Danchin and Fang (2016) lead to the assignment of a significant number of functions to these unknown elements, belonging to core cellular processes such as DNA replication and cell division (e.g. a membrane protease involved in bacterial division), DNA metabolism (e.g. deoxyribonucloside kinases and phosphatases), RNA metabolism (e.g. ribonucleases, nanoRNAses, and helicases), translation‐related functions (e.g. methyltransferases) and functions within general metabolism (e.g. enzymes involved in redox balance, peroxiredoxines and ATP‐dependent Fe^2+^ transporters).

In all, the studies above pinpoint, again, the difficulties in *a priori* deciding which cell functions might be dispensable, and constant developments in the field are expected to further clarify this issue in the near future. While most of the available information on reduced‐ and minimal‐genome bacterial *chassis* is restricted to just a handful of species, other strains will be surely added to the metabolic engineering agenda for specific applications soon. The general physiological and metabolic properties, as well as the advantages and the potential shortcomings, of some of the most representative bacterial strains that could constitute relevant *chassis* are described in the next section.

## Bacterial species adopted as a *chassis*: from historical examples to up‐and‐coming additions

**Figure 3:**
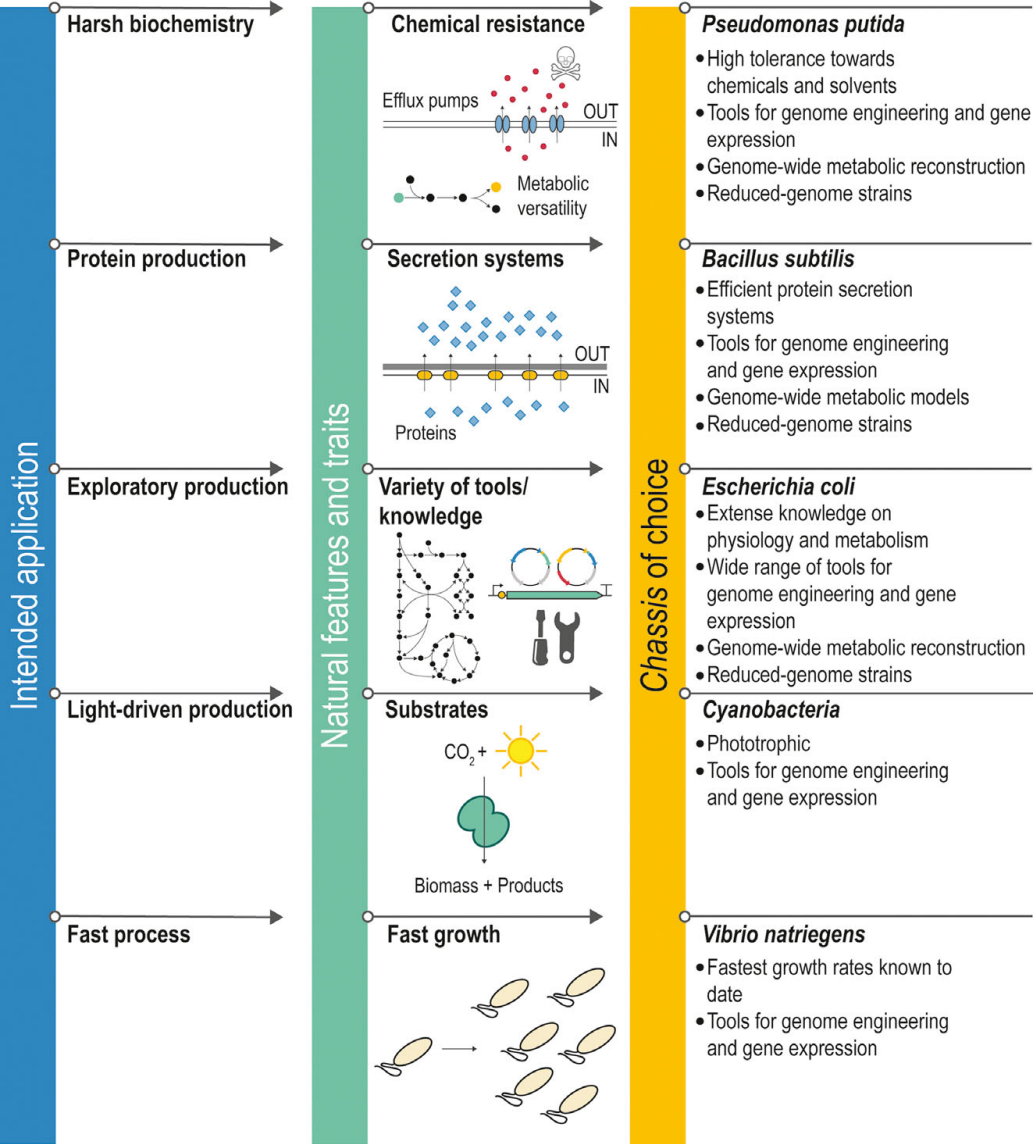
Functional relationship between intended industrially relevant practical applications and different bacterial *chassis* of choice, depending on some key physiological and metabolic features they present. For the sake of simplicity, only some selected applications are shown along with the bacterial species that could be adopted as the starting point for robust *chassis* design and construction. Note that, given the characteristics of some of these bacterial species, they could potentially fulfil more than one application.

Several wild‐type bacteria have been chosen as *chassis* for bioproduction, from laboratory‐derived *E*. *coli* to (more recently) microbial species isolated because of specifically interesting phenotypic properties. In an attempt to describe the main advantages of these hosts in particular applications, we start off by presenting the state of the art of one of the most used Gram‐negative bacterial *chassis*,* E. coli*, and one Gram‐positive bacterium that has been likewise used extensively, *Bacillus subtilis*. Building on their applications, other microorganisms that have gained relevance as a *chassis* for metabolic engineering (e.g. *P. putida*) are discussed at the light of their unique physiological and metabolic properties. For the sake of simplicity, we will focus our discussion mostly on the examples listed above. Other relevant bacterial species that have found important industrial applications, for example *Corynebacterium*,* Clostridium*, and *Streptomyces*, are collected in Table 1, highlighting some of their unique physiological properties along with key examples from the literature that the reader could access if interested on such platforms. In the sections below, we discuss the most relevant features and advantages of *E*. *coli*,* B*. *subtilis* and *P*. *putida* in relation to metabolic engineering efforts and bioproduction of biochemicals, as well as different examples of industrial and/or laboratory‐scale processes using them as platforms. Figure 3 summarizes the main properties of these bacterial *chassis*, listing the practical applications in which they are known to excel.

**Table 1:** Examples of bacterial platforms endowed with unique physiological and metabolic properties for bioproduction.^a^

Microorganism	Advantages	Main type of products obtained	References
*Clostridium acetobutylicum*	Suitable for anoxic bioprocesses, good solvent tolerance, grows on several feedstocks	Acetone, butanol, and butanol, and other solvents in engineered strains	(Ni and Sun, 2009; Jang *et al*., 2012)
*Streptomyces* sp.	Wide variety of secondary metabolites and cognate pathways	Antibiotics	(Li and Townsend, 2006; Komatsu *et al*., 2013; Hiltner *et al*., 2015; Palazzotto and Weber, 2018)
*Corynebacterium glutamicum*	Used for industrial production of amino acids for over 60 years	L‐amino acids (e.g. glutamate and lysine), organic acids, diamides	(Becker and Wittmann, 2012; Wendisch, 2014; Heider and Wendisch, 2015; Unthan *et al*., 2015)
*Rhodococcus* sp.	Lignin degradation and high tolerance to toxic compounds	Acrylamide, triacylglycerols	(McLeod *et al*., 2006; Kosa and Ragauskas, 2013; Röttig *et al*., 2016; Sun *et al*., 2016)
*Mycobacterium* sp.	Natural capability of synthesizing and metabolizing sterols	Steroid intermediates	(Fernández‐Cabezón *et al*.,^2018^)

**a**. Selected examples of bioproduction are presented based on the main industrial applications of these bacterial species.

### Escherichia coli

More than 50 years of intensive research on *E. coli* have positioned this bacterium as the best‐studied example among all prokaryotes, and as an obvious first‐choice as a *chassis* for development of cell factories. *E. coli* is a Gram‐negative, rod‐shaped, facultative anaerobic bacterium (although a more proper classification of its lifestyle would be as a *facultative aerobe*), which can be found in mammalian intestines, some natural environments, and, in some cases, contaminated foods. Its role as a host in biotechnology can be tracked back to its use for l‐threonine production in the 60s (Huang, 1961), and its application for insulin production (Riggs, 1981). It is characterized by (i) rapid growth rates, (ii) low nutrient requirements (which lead to likewise reduced production costs), (iii) the possibility of stablishing high‐cell‐density cultures through fed‐batch fermentation (Shiloach and Fass, 2005), (iv) a versatile metabolism that has been thoroughly investigated and (v) a variety of tools for genetic manipulations and strain development. Although some strains are known to be pathogenic, many of them are safe to use for bioproduction. *E. coli* strains most commonly used nowadays are based on the K‐12 and B ancestor strains, isolated in 1922 and 1918 respectively (Blount, 2015). From these two original isolates, a wide variety of improved strains has been tailored for specific purposes, for example *E*. *coli* BL21 Rosetta, designed for production of heterologous proteins using tRNAs that recognize rare codons in mRNA; *E*. *coli* BL21(DE3), containing a copy of the gene encoding the RNA polymerase from phage T7 integrated in the chromosome; or *E*. *coli* C41(DE3) and C43(DE3), characterized by a high tolerance to (usually toxic) membrane protein expression (Miroux and Walker, 1996). A comprehensive list of *E*. *coli* strains adapted for other purposes has been listed by Baeshen *et al*. (2015). In addition, a number of reduced‐genome strains have also been constructed starting from wild‐type *E. coli* strains. Some of them show significant physiological advantages when compared to the parental, K‐12 strain MG1655. This is the case for the strain set dubbed MDS41, 42 and 43 (15% of the bacterial genome deleted), which have high electroporation efficiency and increased stability of foreign, difficult‐to‐clone DNA due to the removal of all insertion sequences (Pósfai *et al*., 2006). Another set of reduced‐genome *E. coli* strains, derived from W3110, were constructed in the context of the *Minimum Genome Factory* project launched in Japan in 2001 (Mizoguchi *et al*., 2007). One such strain, termed MGF‐01 and having 22% of its genome deleted, reached a final cell density significantly higher than the parental strain and it produced twice as much (i.e. 10 g l^−1^) l‐threonine when grown under the same culture conditions.

A vast number of plasmid vectors and gene expression systems can be found among the tools specifically developed for *E. coli*. This (ever expanding) toolbox includes an extensive catalogue of optimized natural and synthetic promoters, libraries of ribosome binding sites and other regulatory elements, plasmidial origins of replication, resistance markers, affinity tags for protein purification and efficient transcriptional terminators (Terpe, 2006; Rosano and Ceccarelli, 2014; Dvořák *et al*., 2015; Marschall *et al*., 2017; Segall‐Shapiro *et al*., 2018). Following the very spirit of synthetic biology, some of these tools have adopted specific standards and protocols followed suit, such as the *Registry of Standard Biological Parts* (Peccoud *et al*., 2008) or the *BioBricks* repository (Røkke *et al*., 2014). Moreover, numerous genome edition tools have been developed, that have enabled fast and easy strain manipulation through insertion and deletion of genetic circuits into the *E. coli* chromosome (Datsenko and Wanner, 2000; Bloor and Cranenburgh, 2006). More recently, the adoption of techniques based on clustered regulatory interspaced short palindromic repeats (CRISPR) in association with Cas9‐based counter selection systems for genome editing (Jiang *et al*., 2013, 2015; Doudna and Charpentier, 2014), deactivated Cas9 protein for targeted gene regulation (*CRISPRi*, CRISPR interference; Qi *et al*., 2013) and multiplex automated genome editing (*MAGE*; Wang *et al*., 2009) have enormously helped to boost our ability to construct engineered *E*. *coli* strains in a straightforward, streamlined fashion – thus increasing the diversity of strains that can be used for specific bioproduction purposes. In addition to these technologies, the discovery, understanding and application of gene expression devices (e.g. riboswitches and aptamers) allow for the fine‐tune regulation of gene expression in complex pathways (Kushwaha *et al*., 2016). Likewise, the development of biosensors has helped in screening and improving the synthesis of both bulk and fine chemicals. Metabolite biosensors, which respond to the presence of a certain molecule with an easily detectable output, provides an easy way of screening for efficient‐producing strains from complex libraries (Zhang and Keasling, 2011; Liu *et al*., 2015). Moreover, a collection of knock‐out mutants (i.e. the *Keio collection*) is available, providing a simple and high‐throughput way for testing phenotypes and functions of single, non‐essential genes (Baba *et al*., 2006). Data integration from deep genome sequencing (including strains from the Keio collection), as well as elaborated *omics* techniques, has led to the development of robust computational models that predict the metabolic behaviour of an organism under certain simulated conditions. Not surprisingly, such *genome‐scale metabolic models* were firstly reconstructed for *E. coli* (Orth *et al*., 2011). All *E*. *coli* metabolic models available up to date, as well as their potential practical applications, have been reviewed by McCloskey *et al*. (2013) and, more recently, by O'Brien *et al*. (2015). The combination of this broad range of dedicated tools, both for *in silico* predictions and wet‐laboratory manipulations, has made it possible to adopt *E*. *coli* as a model bacterial *chassis* for metabolic engineering and synthetic biology – being a veritable workhorse for developing and testing designer metabolic pathways.

The development of even more robust production strains using *adaptive laboratory evolution* (ALE, also known as *evolutionary engineering*) has enabled the production of high amounts of toxic compounds (Shepelin *et al*., 2018). An ALE application studied in great detail is increasing growth efficiency on specific carbon sources, for example glycerol (Herring *et al*., 2006) or glucose (Notley‐McRobb and Ferenci, 1999; LaCroix *et al*., 2015). Moreover, other *E*. *coli* strains have been evolved to utilize alternative carbon sources, for example citrate (Blount *et al*., 2008), which are not naturally consumed by the parental strain. ALE‐mediated adaptation of bacterial *chassis* to industrially relevant toxic compounds as well as by‐products generated by the extant metabolism, which may affect bacterial growth during production, have been likewise demonstrated. Some examples of adaptation to organic solvents yielded *E*. *coli* strains with high tolerance towards ethanol (Goodarzi *et al*., 2010), *iso*‐ and *n*‐butanol (Atsumi *et al*., 2010; Dragosits and Mattanovich, 2013), and ionic liquids (Mohamed *et al*., 2017). Recently, ALE has been applied together with flux balance analysis to generate an *E*. *coli* strain with increased succinate production from glycerol (Tokuyama *et al*., 2018), and l‐serine, an amino acid usually considered to be toxic (Mundhada *et al*., 2017). The potential of ALE for evolving industrially relevant production phenotypes has been mostly limited to *E*. *coli*, and this technique will surely help to establish other bacterial *chassis* for metabolic engineering by generating useful phenotypic traits.

Different *E. coli* platform strains have been developed for boosting the formation of key metabolites of central carbon metabolism that can be then used as key precursors for target compounds. Typical examples of this sort of manipulations include manipulations leading to increased pyruvate or acetyl‐coenzyme A (CoA) levels. These central metabolites can be used as building blocks in recombinant *E*. *coli* for small molecules such as butanol (Shen *et al*., 2011), or more complex chemical species such as polyhydroxyalkanoates [PHAs (Anjum *et al*., 2016; Chen and Jiang, 2017), specially the simplest form of these polymers, poly(3‐hydroxybutyrate), PHB (Gomez *et al*., 2012)] and fatty acids (Sarria *et al*., 2017). The strategies used for enhancing the formation of such metabolic precursors usually involve deleting reactions that deplete pyruvate or acetyl‐CoA or boosting carbon fluxes through glycolytic pathways (Sánchez‐Pascuala *et al*., 2017). Such metabolic engineering strategies, recently reviewed by Matsumoto *et al*. (2017), not only encompass the manipulation of target structural genes but also global regulators of metabolism, such as the ArcAB two‐component system (Bidart *et al*., 2012). These platform *E*. *coli chassis* characterized by an increased availability of key metabolites can be further used to express a wide variety of heterologous pathways tailored to produce heterologous compounds that are not part of the extant biochemical network. The production of aromatic compounds constitutes a traditional example of this type of metabolic manipulations (Martínez *et al*., 2015). Structural precursors for aromatic compounds are produced in bacteria through the shikimate pathway, which starts with the condensation of phosphoenolpyruvate (PEP) and erythrose‐4‐phosphate (E4P). The shikimate pathway is an essential part of the extant metabolism, serving as the precursor of the aromatic amino acids tyrosine, tryptophan and phenylalanine. Apart from their intrinsic value as food additives, these amino acids can be converted into relevant added‐value compounds, for example vanillin or *p*‐hydroxystyrene (Lee and Wendisch, 2017), or can be used as drug precursors. Other intermediates within the shikimate pathway can redirected to the synthesis of phenol and *cis*,*cis*‐muconic acid. The formation of the key metabolites PEP and E4P has been manipulated in order to increase the overall flux through the shikimate route, and, in a recent example, these manipulations lead to a significant increase in salicylic acid formation (Noda *et al*., 2016). Another strategy adopted for this purpose has been to relieve the feedback regulation that occurs in one of the first enzymes of the pathway, AroF. The *aroF* gene encodes a 3‐deoxy‐D‐arabino‐heptulonase (DHAP) synthase, and a single mutation, leading to an amino acid change in position 148 of the protein (AroF^P148L^), generated the feedback‐resistant *aroF* ^*fbr*^ variant (Weaver and Herrmann, 1990). This specific AroF variant has been used in many studies to boost the shikimate pathway for production of aromatic compounds (Sengupta *et al*., 2015). Targeted deletion of some native genes involved in amino acid synthesis to remove competition with the heterologous enzymes has also been reported, although these manipulations often result in altered growth or significant increases in the process cost, as some amino acids and vitamins have to be supplemented to the culture medium (Noda *et al*., 2016). Another approach, recently implemented to overcome nutritional deficiencies in engineered *E*. *coli* strains, includes transcriptional down‐regulation of *aroK* (shikimate kinase, which consumes shikimate) using growth‐phase dependent promoters (Lee and Wendisch, 2017).

Another family of products of great interest in the field of metabolic engineering are those derived from the mevalonate (MVA) pathway. MVA is the main precursor of a range of added‐value products such as terpenoids and isoprenoids, through the key intermediates isopentenyl pyrophosphate (IPP) and dimethylallyl diphosphate (DMAPP). These compounds found wide applications in the fields of therapeutics, cosmetics, biofuels, and as colour and fragrance (food) additives. Although IPP and DMAPP can be synthesized endogenously by *E. coli* through the methyleythritol‐4‐phosphate (MEP) pathway without generating MVA; this route has been shown to have a very limited flux (Ajikumar *et al*., 2010). Even though the endogenous MEP pathway is less efficient than expressing the heterologous MVA pathway (Morrone *et al*., 2010), quite some work has been devoted to optimize its performance and further understand its wiring (Kim and Keasling, 2001; Zhou *et al*., 2012; Bongers *et al*., 2015). The emerging picture indicates that further optimization is needed when the MVA pathway is used to release metabolic bottlenecks and reduce toxicity of some metabolic intermediates therein (Martin *et al*., 2003; Ajikumar *et al*., 2010; Ma *et al*., 2011; Wang *et al*., 2016a). The adoption of alternative bacterial *chassis* could certainly be a way forward to overcome some of these limitations.

Industrially, *E. coli* has also been used for the production of 1,3‐propanediol by DuPont and 1,4‐butanediol by Genomatica (Sabra *et al*., 2016). 1,3‐Propanediol, a monomer for the synthesis of industrial polymers such as polytrimethylene terephthalate, can be naturally produced from glycerol by some microorganisms. In order to construct a high‐yield *E. coli* producer, the genes encoding all the functions needed for 1,3‐propanediol synthesis were cloned from *Klebsiella pneumoniae* (Gatenby *et al*., 1998), followed by several metabolic engineering strategies on the resulting strain to ensure sufficient precursor availability. 1,4‐Butanediol, a structurally similar diol, is another bulk chemical used as precursor of industrially‐important plastics and spandex fibres. A library of heterologous genes needed for 1,4‐butanediol formation from succinyl‐CoA, intermediate of the tricarboxylic acid cycle, has been tested in *E. coli* (Yim *et al*., 2011). This early attempt was soon combined with sophisticated metabolic engineering approaches, resulting in a set of *E*. *coli* strains that achieved yields of 1,4‐butanediol – high enough to warrant commercialization. The sequential process for construction and optimization of these engineered *E*. *coli* strains has been recently reviewed in Burgard *et al*. (2016).

All our extensive knowledge and range of tools for engineering *E. coli* make this enteric bacterium a suitable choice as a *chassis* for bulk product formation as well as using it as a proof‐of‐concept for designer (synthetic) pathways at their initial stages of development. There is, however, a limit in the potential uses of *E. coli*, which is not a suitable platform for all bioprocesses and chemicals in the currently expanding bioproduction agenda. Some examples of challenging processes are the production of secreted proteins at high yields or the production of highly toxic compounds, which negatively affect the growth and catalytic efficiency of *E. coli*. Moreover, new and cheaper (raw) alternatives to traditional substrates are required in order to minimize production costs. The utilization of different bacterial *chassis*, described below, attempts to overcome these challenges.

### Bacillus subtilis

*Bacillus subtilis* is an aerobic, rod‐shaped, Gram‐positive bacterium found in soils and in the plant rhizosphere. It is one of the best known and characterized Gram‐positive microorganisms, due to a number of early studies on its natural competence for DNA transformation and the formation of spores as resistant, non‐reproductive structures (Spizizen, 1958). The genome of *B*. *subtilis* strain 168 was one of the first to be sequenced (Kunst *et al*., 1997), and a number of tools for genome manipulation have been developed ever since, as well as a genome‐scale reconstruction of its metabolic network 10 years after the genome sequence became available (Oh *et al*., 2007). More recently, a full collection of knock‐out mutants of *B. subtillis* has been developed (Koo *et al*., 2017). Importantly, *B. subtilis* is free of endotoxins and has the generally recognized as safe (*GRAS*) status as a microorganism for protein production. The availability of numerous genetic tools, such as vectors and gene expression systems, makes it an easy‐to‐handle organism (Liu *et al*., 2017a). As mentioned in the preceding sections, there has been extensive work in constructing reduced‐genome versions of *B. subtilis*. Among them, the reduced‐genome strain MGB874 has been shown to produce higher concentrations of alkaline cellulose and protease as heterologous proteins (Morimoto *et al*., 2008; Manabe *et al*., 2013).

Recombinant proteins and natural enzymes and proteases constitute the main portfolio of industrially relevant molecules produced in *B. subtilis chassis* – essentially because its efficient secretory machinery allows for the transport of proteins into the culture medium to reach concentrations in the range of grams per litre, thus reducing purification and recovery costs (van Dijl and Hecker, 2013). The adequate signal peptides are required in the target proteins to ensure a correct and efficient secretion. Such signal peptides are composed by three domains, known as N‐, H‐ and C‐ regions, recognized by different secretory machineries for the proper translocation of proteins from the cytoplasm through the membrane and to the external medium. The best‐studied protein secretory systems in *B. subtilis* are the Sec and Tat pathways (Hohmann *et al*., 2017). However, *B. subtilis* naturally produces a number of proteases and, although some of them might have an interest for the industry on their own, their activity usually limits the overall efficiency of heterologous protein production. For this reason, the construction of proteases‐defective mutants has been crucial for the development of *B*. *subtilis* as a *chassis* for protein production. Some of the most used mutants of this sort are the type strains WB600 (Wu *et al*., 1991) and WB800 (Wu *et al*., 2002), in which six and eight proteases, respectively, have been eliminated.

Furthermore, the development of *B. subtilis* as a *chassis* for heterologous protein production has included the design of a wide variety of gene expression systems. Different types of strong, constitutive as well as inducible promoters used in *B. subtilis* have been reviewed by Song *et al*. (2015). The *Bacillus* Genetic Stock Centre (BGSC) was created for maintenance and distribution of the (ever increasing) catalogue of characterized *Bacillus* strains and knock‐out mutants. Apart from the strains, the BGSC collection also contains cloning vectors and expression plasmids that can be used in *B. subtilis*. However, some of these plasmids tend to be unstable and, due to the natural ability of *B. subtilis* of DNA uptake and integration into the chromosome through double crossover, homologous recombination, single‐copy DNA integration is the most used method for heterologous gene expression (Kunst and Rapoport, 1995), although novel and easy ways for DNA transformation and mobilization are being developed (Miyano *et al*., 2018). Several genomic regions in the *B*. *subtilis* chromosome have been characterized for heterologous gene expression; such as *amyE* (encoding an α‐amylase) which provides a coloured‐colony phenotype by performing an α‐amylase test to check for successful gene integration. This strategy has been adopted by Commichau *et al*. (2014) for engineering efficient vitamin B6 production in *B*. *subtilis* strain 168.

As described before, *B. subtilis* is a very attractive organism for enzyme production, as their easy secretion outside the cell simplifies the downstream processing as well as their (re)folding when needed (Westers *et al*., 2004). The global industrial production of enzymes market is expected to reach $ 6.2 billion in 2020, and these enzymes have applications as varied as production of detergents, treatment of textiles, additives for the food industry, cosmetics and waste degradation (Singh *et al*., 2016). Some examples of industrially relevant enzymes produced in *B. subtilis chassis* are subtilisin (an alkaline serine protease), α‐ and β‐amylases, β‐glucanases and laccases (Schallmey *et al*., 2004). Numerous examples on the optimization of heterologous enzyme production in *B*. *subtilis* have been published – which attempt to improve the overall process efficiency, finding new interesting enzymes and to develop more suitable *chassis* for the industry (Chen *et al*., 2016; Feng *et al*., 2017).

Apart from its prominent role in protein production, *B. subtilis* is also used for industrial processes aimed at the synthesis of nucleotides, vitamins, surfactants and antibiotics, for example bacitracin and subtilin. Nucleotides, for example inosine monophosphate (IMP) and guanosine monophosphate (GMP), are extensively used in a wide range of processed foods as flavour boosters in combination with monosodium L‐glutamate. Industrial production of these nucleotides has traditionally relied in Gram‐positive microorganisms and, among them, *B. subtilis* and other *Bacillus* species have a prominent role due to a large accumulation of inosine in the culture medium (Chen *et al*., 2005). Building on this natural occurrence, multiple studies have been performed to improve nucleotide accumulation, using strategies such as classical random mutagenesis (Matsui *et al*., 1982), culture optimization (Chen *et al*., 2005), target mutagenesis to avoid IMP degradation (Asahara and Mori, 2010) or introduction of nucleotidases that remove the phosphate group from IMP and GMP (Terakawa *et al*., 2016). A number of vitamins are also being produced industrially in *B*. *subtilis* as the *chassis*, mainly riboflavin, cobalamin and biotin. Riboflavin, a component of the vitamin B2 complex, is a major nutrient in human diet, which can be naturally found in vegetables and raw milk. Nowadays, riboflavin is produced by fermentation for its use as an additive in the food industry, and *B. subtilis* is one of the microorganisms most often used in such bioprocess. A limitation in the availability of precursor nucleotides (i.e. GTP), however, is known to hinder riboflavin production. For this reason, a deregulation of purine synthesis has been implemented to ensure efficient accumulation of this vitamin (Stahmann *et al*., 2000; Shi *et al*., 2014).

Poly‐γ‐glutamic acid is a homopolyamide naturally produced by *B. subtilis* and related bacteria. A relevant microbial polymer used in food, medical, cosmetic and waste treatment industries (Shih and Van, 2001; Bajaj and Singhal, 2011), it is composed of d‐ and l‐glutamic acid units interconnected through the amide linkage of their α‐amino and γ‐carboxylic groups. The cost‐effective production of poly‐γ‐glutamic acid has been achieved using natural‐producer *Bacillus* strains (Tanaka *et al*., 1997), as well as by improved strains using genome manipulation techniques such as genome shuffling (Zeng *et al*., 2016). *B. subtilis* has also been used to produce pure chiral stereoisomers, for example D‐(—)‐2,3‐butanediol, another promising diol used as a biofuel and bulk chemical for chemical synthesis (Fu *et al*., 2016). Other molecules, for example hyaluronic acid (a high‐molecular‐weight glycosaminoglycan used in the pharmaceutical and cosmetic industry), have been produced in *B. subtilis* up to the range of grams per litre (Jin *et al*., 2016). *Bacillus* has been also applied as a functional *chassis* to produce and secrete a synthetic cellulosome for further use in the degradation of raw cellulosic substrates (Lin *et al*., 2017). The production of other important pharmaceutical molecules, for example *N*‐acetylglucosamine, has been attempted as well, and the bioprocess has been optimized through pathway modulation and successful colocalization of pathway enzymes using scaffold proteins (Liu *et al*., 2017b,c).

All the examples above accredit the value of *B. subtilis* as a robust *chassis*, well established in the biotechnology field for production of endogenous enzymes – and also for the production of heterologous proteins, although the titres are usually not comparable to those of endogenously synthesized proteins. For these reasons, more research needs to be performed in this bacterial strain, including genome reduction, identification and optimization of signal peptides, development of novel, more efficient secretion and expression systems, and further optimization of both culture settings and resistance to stressful conditions (Tjalsma *et al*., 2004; Song *et al*., 2015; Oztürk *et al*., 2016). In bioprocesses for which *built‐in* resistance to harsh operating conditions is needed, the focus shifts to a different type of bacterial *chassis*, as discussed in the next section.

### Pseudomonas putida

Chemical stress, for example under the form of either endogenously produced or exogenously added chemicals, is one of the main hurdles encountered in industrial bioprocesses. Either the desired product or some of the substrate feedstocks (e.g. some compounds found in biomass hydrolysates) can inhibit bacterial growth or even cause cell death (Keasling, 2010). In both cases, the yields and titres of the bioprocess are negatively affected, leading to sub‐optimal production performance. Moreover, two‐phase fermentations, where a second organic phase is used to extract the product from the aqueous phase [e.g. during production of *p*‐vinylphenol or 1,3‐propanediol (Rujananon *et al*., 2014; Salgado *et al*., 2014)], require the use of microorganisms able to tolerate the solvent(s) used as second phase. Specifically, due to toxic effects, the choice of solvents for whole‐cell biotransformations in two‐phase solvent‐water systems is usually very limited. Only low‐toxicity solvents with high hydrophobicity coefficients can be applied for this purpose, limiting the scope of possible bioprocesses that can be carried out under these conditions. For these reasons, the adoption of bacterial *chassis* with an enhanced tolerance to chemical stresses is needed (Nicolaou *et al*., 2010; Kusumawardhani *et al*., 2018).

The ubiquitous saprophytic, soil‐colonizer *P. putida*, a Gram‐negative, rod‐shaped bacterium is increasingly being used as a *chassis* for applications characterized by harsh operating conditions. *P*. *putida* KT2440, which has been certified as a GRAS platform for recombinant protein production, is the most studied and used strain within the genus, and it is considered a safe host for cloning and expressing heterologous genes (Poblete‐Castro *et al*., 2017). *P. putida* KT2440 possesses many of the desired features in an ideal bacterial *chassis*, such as rapid growth, low nutritional requirements and availability of sophisticated tools for genome and genetic manipulation (Nikel and de Lorenzo, 2018). A collection of mutant strains obtained by random integration of mini‐Tn*5* elements, known as the *Pseudomonas* Reference Culture Collection, is available for strain KT2440 (Duque *et al*., 2007). Moreover, this soil bacterium is endowed with built‐in, advantageous evolutionary traits, for example a remarkably versatile metabolism that serves as a treasure trove for enzymatic activities, and increased tolerance towards oxidative stress. *P. putida* can also sustain high rates of NADPH regeneration when growing on hexoses, due to the fact that glucose is converted into glyceraldehyde‐3‐phosphate and pyruvate *via* the Entner‐Doudoroff (ED) pathway (Martins dos Santos *et al*., 2004; del Castillo *et al*., 2007; Nikel *et al*., 2015a), and part of these trioses‐phosphate are recycled back into hexoses‐phosphate, generating one molecule of NADPH *via* a combination of activities of the ED pathway, the pentose phosphate pathway, and an incomplete Embden‐Meyerhof‐Parnas route, collectively termed *EDEMP cycle* (Nikel *et al*., 2016). This particular metabolic architecture has been shown to enable a better expression of heterologous enzymatic pathways and serves as an efficient source of reducing power for maintaining a high tolerance to stressful conditions (Blank *et al*., 2008; Chavarría *et al*., 2013; Nikel *et al*., 2014b). Furthermore, *P. putida* KT2440 is able to use a wide range of compounds as carbon sources, such as succinate, citrate and other intermediates of the tricarboxylic acid cycle. More importantly, the original soil environment of *P*. *putida* KT2440 and its ability to thrive in the rhizosphere are connected with its ability to degrade aromatic compounds derived from lignin degradation, for example benzoate, *p*‐coumarate, caffeate and vanillate (Jiménez *et al*., 2002; Dvořák *et al*., 2017). This fact makes *P. putida* KT2440 an important candidate for its use to grow in lignin‐derived feedstocks (Linger *et al*., 2014; Ragauskas *et al*., 2014; Beckham *et al*., 2016; Ravi *et al*., 2017).

Remarkably, *P. putida* has a number of mechanisms for tolerance towards high concentrations of aromatic chemicals, for example toluene, xylenes and styrene (note that these toxic compounds can also be used by some *P*. *putida* strains), including a wide repertoire of efflux pumps (Inoue and Horikoshi, 1989; Ramos *et al*., 2002; Santos *et al*., 2004; Domínguez‐Cuevas *et al*., 2006; Calero *et al*., 2018). Toluene, for instance, is an aromatic and highly toxic solvent that kills most microorganisms at concentrations as low as 0.1% (v/v). Being an industrial feedstock, it kept accumulating in the environment since its very discovery back in the 19th century. Several efforts were therefore aimed at finding microorganisms able to degrade toluene, converting it into less‐harmful compounds. Even when some *Achromobacter* and *Nocardia* species were known to tolerate moderate concentrations of toluene, a true robust and resistant strain chasing was largely missing in the picture for many years. The seminal work by Prof. Horikoshi's group in the late 80s in this regard showed that an isolate of *Pseudomonas* sp. could thrive in the presence of very high concentrations of toluene. Soon after the publication of this article, Prof. de Bont's group in The Netherlands and that of Prof. Ramos in Spain reported the isolation of two other *Pseudomonas* strains that were also able to grow in the presence of saturating concentrations of toluene. A most surprising finding was that all three strains isolated in the three countries and different niches happened to be the same microorganism, *P*. *putida*. The discovery of solvent tolerance opened a new research avenue and aroused great interest in the use of this kind of bacterial *chassis* in bioremediation and biotransformations in biphasic systems, as well as in the development of biosensors for environmental contaminants. How do *Pseudomonas* cells thrive under these harsh conditions? As indicated above, the most relevant of the many mechanisms for solvent tolerance in *Pseudomonas* seems to be related to the action of a series of efflux pumps that extrude toluene (and, likely, other solvents) from the cell membranes to the outer medium, a high energy‐demanding process whose price bacteria are forced to pay to survive under such extreme conditions. Another important mechanism that contributes to solvent tolerance involves changes in phospholipid composition, that is isomerization of *cis*‐unsaturated fatty acids to *trans*‐isomers, and changes in head group composition in membrane phospholipids. All these changes influence membrane fluidity and consequently have an effect on resistance to the membrane chaos brought about by solvents. Apart from lipid disorganization, protein unfolding occurs when toluene and other solvents dissolve in cell membranes. This situation brings forth a general stress response on bacteria that, in most cases, is accounted for by expressing several molecular chaperones and other enzymes related to stress resistance.

Tolerance to solvents, and the ability of using them as carbon sources, is a very oxygen‐demanding cellular process. *P. putida* KT2440 is a strict aerobe due to the absence of fermentative pathways and the inability of using alternative electron acceptors other than oxygen – leading to a somewhat limited number of naturally synthesized by‐products under industrially relevant conditions (Martínez‐García *et al*., 2014b; Tiso *et al*., 2014). The lack of fermentation pathways also leads to a very high oxygen requirements for optimal bacterial growth, which can be a problem when culturing bacteria in large bioreactors giving the non‐homogenous distribution of nutrients (Davis *et al*., 2015), as well as impairing the practical use of *P. putida* KT2440 to carry out anoxic, oxygen‐sensitive reactions. This problem was assessed by introducing synthetic fermentation pathways that, when expressed in strain KT2440, lead to higher survival under anoxic conditions (Nikel and de Lorenzo, 2013). Schmitz *et al*. (2015) and Lai *et al*. (2016) also tackled this problem by employing bioelectrochemical systems. This is an area of intense research currently under development, which aims at modifying the lifestyle of environmental bacteria, and holds the promise of further multiplying the uses of *P*. *putida* as a functional *chassis* under a range of operating conditions.

A number of tools for genetic and genome engineering of *P. putida* have been developed, including the complete set of modular vectors of the *Standard European Vector Architecture* (SEVA) platform (Silva‐Rocha *et al*., 2013; Martínez‐García *et al*., 2015), transposons (Martínez‐García *et al*., 2014a) and a wide range of promoters, both natural and synthetic, which have been characterized in strain KT2440 (Zobel *et al*., 2015; Calero *et al*., 2016). Homologous recombination‐based techniques (e.g. using the homing endonuclease I‐*Sce*I from *Saccharomyces cerevisiae* that recognizes an 18‐bp DNA sequence, not present in bacterial chromosomes) have been implemented for deleting large genomic fragments (Martínez‐García and de Lorenzo, 2011). The procedure is based on forcing homologous recombination by the appearance of a double strand break in the target genome upon cleavage *in vivo* by I‐*Sce*I, the intracellular expression of which is driven by the 3‐methylbenzoate‐inducible promoter *Pm* in a broad host range expression plasmid. Using this system, 69 genes involved in synthesis and functioning of the flagellar machinery were successfully deleted from strain KT2440 (Martínez‐García *et al*., 2014c), as well as a number of genes related to genetic instability, such as insertion sequences and transposons (in total, 4.3% of the genome DNA was eliminated). These operations generated a reduced‐genome *P. putida chassis*, EM42, which showed a significant increase in ATP and NAD(P)H availability. The growth of the resulting *chassis* showed improvements in both rich and minimal medium with different carbon sources, with enhanced resistance to reactive oxygen species, which in turn led to an increased heterologous GFP and luciferase production (Martínez‐García *et al*., 2014b). In further tests conducted in bioreactors, the reduced‐genome *chassis* had significantly improved plasmid stability and heterologous protein production, among other traits, as compared to the parental strain (Lieder *et al*., 2015). Novel tools for advanced genome edition, based on the expression of specific DNA recombinases, are constantly being developed for *P. putida* KT2440 (Martínez‐García and de Lorenzo, 2017), for example for increasing the efficiency of DNA recombination. The development of these techniques will allow for the use of precise MAGE and CRISPR/Cas9‐based technologies in the near future, speeding‐up genomic manipulations in *Pseudomonas chassis*.

In addition to the genetic and genome engineering tools, a number of bioinformatic tools have been developed to facilitate the rational design of metabolically engineered strains based on *P. putida* KT2440. Soon after the sequencing of its entire genome in 2002 by Nelson *et al*. (2002), three genome‐scale metabolic models were developed for strain KT2440 (Nogales *et al*., 2008; Puchałka *et al*., 2008; Sohn *et al*., 2010) – with a recent update on the occasion of the genome re‐sequencing and re‐annotation (Belda *et al*., 2016), and the most comprehensive genome‐wide metabolic reconstruction build to date (Nogales *et al*., 2017). The integration of *in silico* model predictions and experimental data from *omics* (e.g. deep RNA sequencing) has provided further insights into the metabolism of *P. putida* KT2440 (Kim *et al*., 2013; Nikel *et al*., 2014a). Finally, a number of databases are currently available, with continuously updated genetic information (e.g. the *Pseudomonas* Genome Database; Winsor *et al*., 2016) and a protein–protein interaction database (*PutidaNET*; Park *et al*., 2009). A platform integrating all the *omics* information available for *P*. *putida* would be highly desirable, facilitating further manipulations of an already attractive bacterial *chassis*.

Although the seminal works on the practical biotechnological applications of *P. putida* have been focused on xenobiotics degradation, the use of this *chassis* as a bacterial cell factory for bioproduction increased exponentially over the last decades (Poblete‐Castro *et al*., 2012; Loeschcke and Thies, 2015; Nikel *et al*., 2016). One of the most well‐known products synthesized using *Pseudomonas* species is the family of PHAs polyesters (Prieto *et al*., 2016). PHAs are naturally synthesized by *P. putida* under specific conditions, for example nitrogen‐limited conditions in the presence of sufficient amounts of a suitable carbon source (Hoffmann and Rehm, 2004), and these polyesters are used as carbon and energy storage. The biodegradability of PHAs as well as their material properties, such as thermoplasticity, insolubility and lack of toxicity, make them good alternatives for fuel‐based plastics for ecofriendly packaging and other industrial purposes (Steinbüchel and Lütke‐Eversloh, 2003; Ouyang *et al*., 2007). In addition to the biosynthesis of PHA biopolymers (for which strain Gpo1 has been used at the industrial scale), *P. putida* is gaining importance as a cell factory for *de novo* biosynthesis of heterologous, often difficult‐to‐produce chemical compounds. For many of the target chemicals in this family, the natural tolerance of *P*. *putida* towards aromatic molecules and its ability to convert these chemical structures *via* mono‐ and di‐oxygenases (Pérez‐Pantoja *et al*., 2013) have been exploited for strain engineering. Relevant cases of this sort include the synthesis of 3‐methylcatechol (Hüsken *et al*., 2001), *o*‐cresol (Faizal *et al*., 2005), *cis,cis*‐muconate (van Duuren *et al*., 2011, 2012) and styrene (Blank *et al*., 2008).

Other compounds produced in *P. putida* are rhamnolipids, low toxic, biodegradable bacterial biosurfactants, *via* the heterologous expression of the *rhl* genes from the *Pseudomonas aeruginosa* biosynthesis pathway (Wittgens *et al*., 2011; Tiso *et al*., 2017). *P. putida* has also been used as a functional *chassis* for the production of terpenoids, taking advantage of the natural stress resistance of this species to toxic chemicals. Production of (*S*)‐perillyl alcohol, for instance, has been achieved by expressing a cytochrome P450 (van Beilen *et al*., 2005); and *de novo* production of geranic acid has been engineered by coexpressing a geraniol synthase from *Ocimum basilicum* together with genes encoding the MVA pathway for isoprenoid synthesis (Mi *et al*., 2014). Zeaxanthin is another class of terpenoid that has been efficiently produced in *P. putida* (Beuttler *et al*., 2011; Loeschcke *et al*., 2013). Moreover, a number of aromatic compounds have been produced in *P. putida* using the amino acids derived from the shikimate pathway as precursors. For the production of such molecules, a small number of genes were introduced into the engineered strains, and further metabolic engineering to increase the availability of amino acid precursors was performed in order to improve yields. Interestingly enough, the regulation of these pathways in *P*. *putida* KT2440 and S12 (a solvent tolerant strain) seems to differ from that in *E*. *coli*, which requires the adoption of specific metabolic engineering strategies. Examples of production of added‐value aromatic molecules in *P*. *putida* are illustrated by cinnamic acid (Nijkamp *et al*., 2005) and phenol (Wierckx *et al*., 2005; Wynands *et al*., 2018) – achieved, among other manipulations, by introducing a phenylalanine‐ammonia lyase from *Rhodosporidium toruloides* and the tyrosine phenol lyase from *Pantoea agglomerans*. Other strains producing derivatives of these aromatic compounds were developed, producing, for example *p*‐hydroxystyrene (Verhoef *et al*., 2009), *p*‐hydroxybenzoate (Yu *et al*., 2016), anthranilate (Kuepper *et al*., 2015), vanillate (Graf and Altenbuchner, 2014) and *p*‐coumaric acid (Calero *et al*., 2016). Furthermore, a number of molecules are being synthesized in industrial‐scale fermentations using engineered *P. putida* strains. Such examples include the synthesis of 5‐cyanopentanamide by DuPont (USA) or 5‐methylpyrazine‐2‐carboxylic acid by Lonza (Switzerland) (Kiener, 1992; Tiso *et al*., 2014).

In all, *P. putida* is considered as one of the most promising *chassis* for handling the synthesis of difficult‐to‐produce chemicals, involving harsh reactions and complex biochemistries that impose a high level of chemical stress to the host cells. The broad, rich metabolism of *P*. *putida* also makes it a suitable candidate for the use of cheap feedstock substrates with high levels of impurities, for example lignocellulosic biomass hydrolysates, although more research is still required to increase growth rates and product yields and titres under these operating conditions. While all these issues are actively being investigated nowadays, the next relevant question pertains to the adoption of novel bacterial species that could also serve as the starting point for the construction of robust *chassis*.

## Emergent bacterial *chassis*

Microbial diversity provides a phenomenal source of solutions to the practical problems encountered in metabolic engineering. Screening through the natural biological repertoire of solutions and exploit them for bioproduction must be part of the engineering agenda (Price *et al*., 2018). Some of the current challenges are (i) limitations in growth rates and product yields in engineered bacteria, (ii) minimizing production costs (i.e. feedstock prices) and (iii) simplifying downstream processing. Against this background, in this section, we discuss up‐and‐coming bacterial *chassis* that have exceptionally attractive features to develop them as hosts for bioproduction. The selected examples include *Vibrio natriegens* (endowed with a remarkably fast growth), cyanobacteria (due to their photosynthetic capabilities) and *Roseobacter* and *Halomonas* as marine bacterial species with an unique tolerance to saline stress. Other relevant, emerging bacterial species that could be potentially developed as *chassis* for specific applications include both *Shewanella* and *Geobacter* sp., which can be used to engineer *microbial fuel cells* due to their electron‐accepting capabilities; *Klebsiella* sp., which can produce a variety of low‐molecular‐weight bulk products using glycerol as carbon source; and *Deinococcus*, due to its high resistance to DNA damage and broad range of feedstock utilization (Fredrickson *et al*., 2008; Dantas *et al*., 2015; Gerber *et al*., 2015; Kumar and Park, 2018). Although significant research efforts have been invested on alternative bacterial *chassis* in recent years, much work is still lying ahead to place them at a similar state of acceptance and widespread use as *E. coli* or *B. subtilis*. These challenges notwithstanding, in this section we discuss the state of the art and developments to move the field of metabolic engineering forward by adopting non‐traditional bacterial *chassis*.

### Vibrio natriegens

One of the desired traits of the ideal *chassis* to be used as a cell factory for the production of biomass‐associated products is the fastest growth possible with the lowest nutrients need. *V. natriegens* is a Gram‐negative, facultative anaerobic bacterium. Formerly known as *Pseudomonas natriegens* and *Beneckea natriegens,* it was firstly isolated and described in 1961 (Eagon, 1961; Payne *et al*., 1961). This bacterium has the shortest doubling time known to date: 15 min in brain heart infusion medium supplemented with KCl, MgCl_2_ and additional NaCl at 37°C, and 9.8 min in the same culture conditions but with a culture medium supplemented with sea salt. These doubling times are around two times shorter than those of a fast‐growing *E. coli* strain (e.g. NEB Turbo™) grown in the same culture conditions. The genome of *V*. *natriegens* has been recently sequenced (Maida *et al*., 2013; Lee *et al*., 2016) and, in an attempt to develop this host as a functional *chassis* for bioproduction, a number of techniques and tools have been tested and developed, including expression vectors, protocols for DNA transformation, synthetic promoters covering a range of expression strengths and tools for manipulating gene expression levels *via* CRISPRi (Lee *et al*., 2016; Weinstock *et al*., 2016). The fast growth of this bacterium allows for a significant speed‐up of laboratory cloning procedures, as colonies are already visible on a plate after a mere six‐h post‐transformation incubation. *V. natriegens* was also found to have a secretory system able to export proteins to the culture medium (Weinstock *et al*., 2016). Moreover, natural transformation and multiplex genome editing was achieved by overexpressing the *tfoX* gene from *V. cholera* and *V. natriegens*, achieving up to four simultaneous scarless genome edits (Dalia *et al*., 2017). Further research on the central carbon metabolism of *V. natriegens* has been performed in glucose‐grown cells using ^13^C‐based metabolic flux analysis, showing a flux distribution in central carbon metabolism similar to that of *E. coli* (Long *et al*., 2017). Its suitability to be grown in large bioreactor cultures using different carbon sources has been also tested, achieving cell densities around 20 g cell dry weight l^−1^, and rates of substrate consumption faster than those of *E. coli* and *S. cerevisiae* have been reported under these growth conditions (Hoffart *et al*., 2017). *V. natriegens* has been successfully applied for the production of industrially relevant chemical compounds such as PHAs and L‐alanine, hinting a great potential for biotechnological applications in the near future (Dalia *et al*., 2017; Hoffart *et al*., 2017).

### Cyanobacteria

Cyanobacteria are increasingly becoming attractive *chassis* to sustain production of biofuels and chemicals due to their ability to use sunlight through photosynthesis, which provides a renewable, cheap, and almost unlimited energy source. Since CO_2_ is fixed during the process, a significant reduction of production costs related to feedstocks is also expected. In contrast to other photosynthetic organisms, such as plants, cyanobacteria are characterized by a relatively fast growth (Carroll *et al*., 2018). Several strains of cyanobacteria have been used so far for metabolic engineering, for example *Synechocystis* sp. PCC 6803, *Synechococcus elongatus* PCC 7942, and *Synechococcus* sp. PCC 7002, the genomes of which have been sequenced (Kaneko *et al*., 1995). A number of tools for genome engineering have been developed for these cyanobacterial strains (in particular, for *Synechocystis* sp. PCC 6803 and *Synechococcus elongatus* PCC 7942), and a suite of transformation techniques have been likewise tested, for example *via* natural transformation or electroporation (Wang *et al*., 2012; Yu *et al*., 2013). Neutral insertion genome regions in the chromosome and fluorescent reporters have been studied (Ruffing *et al*., 2016). Moreover, genetic engineering of a *Synechococcus* strain has been achieved using the CRISPR/Cas9 system, although expression of the gene encoding Cas9 brought about severe growth impairment (Wendt *et al*., 2016). Nonetheless, efficient gene repression of several genes has been implemented using CRISPRi in *Synechocystis* sp. PCC 6803 (Kaczmarzyk *et al*., 2018). Cyanobacteria have attracted a lot of attention for the production of biofuels as an alternative to other photosynthetic systems like plants. Apart from the obvious differences in replication rates, the use of cyanobacteria has been proposed as an alternative to overcome the problem of competition with croplands for food, while still avoiding the need of using sugars as feedstock (Ducat *et al*., 2011; Nozzi *et al*., 2013; Savakis and Hellingwerf, 2015). Although cyanobacteria have found a somewhat limited use in industry thus far, several added‐value compounds have been produced using this *chassis*. A classic example of this sort is represented by several biofuels, for example ethanol, bisabolene, farnesene, 1‐butanol, isoprene and isopropanol (Dexter and Fu, 2009; Lindberg *et al*., 2010; Lan and Liao, 2011; Kusakabe *et al*., 2013; Davies *et al*., 2014; Halfmann *et al*., 2014) – which even led to the creation of the spin‐off company Photanol in the Netherlands for cost‐efficient production of biochemicals using cyanobacteria. Bioproduction of other compounds, for example 1,3‐propanediol using CO_2_ as the only carbon substrate, has been engineered through the use of specific promoters and optimization of culture conditions (Hirokawa *et al*., 2017). Furthermore, a wide range of cyanobacteria strains are able to produce PHAs. 3‐Hydroxybutyrate, the key metabolic precursor of PHB, has also been produced in *Synechocystis* sp. PCC 6803 in an attempt to reduce the high biopolymer production costs associated with traditional, sugar‐based fermentation approaches (Wang *et al*., 2013). Although sufficiently high PHB accumulation has been reached, up to 85.1% of the cell dry weight (Samantaray and Mallick, 2012), the volumetric productivities are still far from being economically competitive (Singh and Mallick, 2018). One of the main reasons for this low productivity is the lack of methods and efficient processes for biomass production at a large, industrial scale and the somewhat limited set of tools available for targeted and fast genome manipulations. Sophisticated metabolic engineering approaches for facilitating CO_2_ and formate fixation have been developed over the last few years (Yishai *et al*., 2017; Cotton *et al*., 2018), and will surely help launching cyanobacteria as functional *chassis* for novel metabolic functions towards sustainable bioproduction.

### Roseobacter and Halomonas

Besides bioproduction, alternative engineering applications call for an alternative *chassis* as well. As proposed by Borg *et al*. (2016), this is the case for the bioremediation of plastic waste in marine environments, a specific process for which marine bacteria would be the most suitable *chassis*. One of the most important clades in marine habitats is *Roseobacter*. These bacteria have been described previously as source of secondary metabolites, for example antibiotics (Martens *et al*., 2007; Brock *et al*., 2014; Wang *et al*., 2016b), and techniques for genome manipulation have been developed (Borg *et al*., 2016; Tang *et al*., 2016). Another case of an alternative, marine *chassis* microorganism is *Halomonas*, which can grow in the presence of high salt concentrations under non‐sterile conditions (Tan *et al*., 2011) – significantly reducing costs associated with sterilization in the fermentation process. Although the toolbox for genetic manipulation of this bacterial species is somewhat limited, advances have been made to overcome this problem, for example the development of constitutive and inducible promoters for targeted gene expression (Yin *et al*., 2014; Zhao *et al*., 2017), construction of knock‐out mutants through homologous recombination stimulated by double‐strand breaks in the bacterial genome (Fu *et al*., 2014), and the implementation of the CRISPRi technology (Tao *et al*., 2017). In terms of practical applications, and by combining all these engineering strategies, *Halomonas* strains have been successfully used for bioproduction of PHB and PHAs copolymers containing 3‐hydroxyvalerate (Fu *et al*., 2014), and the osmocompatible solute ectoine (Chen *et al*., 2017).

## Outlook and the challenges ahead

**Figure 4:**
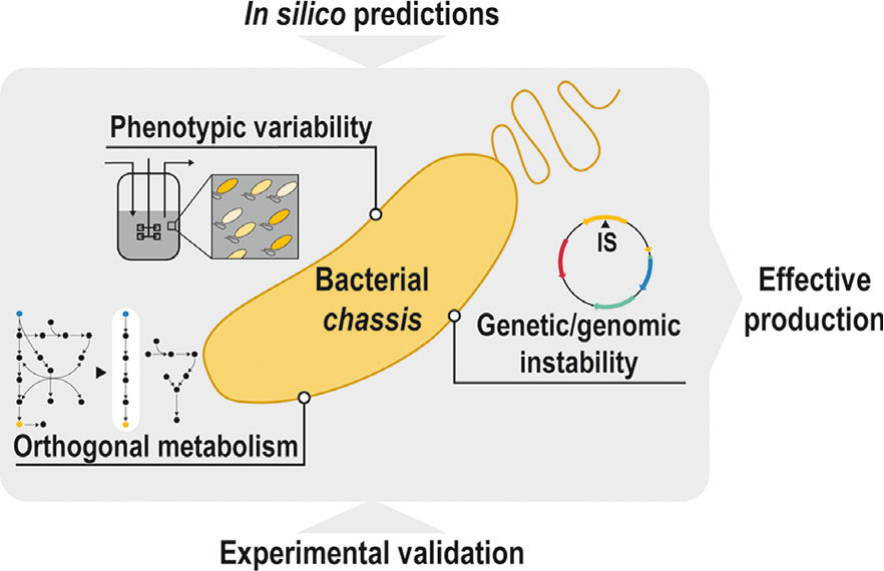
Some of the challenges ahead for developing functional bacterial *chassis* for metabolic engineering. Effective bioproduction could only be achieved by overcoming the current hurdles of genetic and genomic instability (e.g. leading to alteration or loss of production phenotypes), phenotypic variability across individual cells in the microbial population, and the inevitable interactions between metabolic implants and the extant biochemistry of the bacterial *chassis*. The way forward to tackle these issues requires the combination of robust *in silico* predictions and in‐depth experimental validation, if possible under conditions compatible with industrial production.

The preceding sections indicate that, although there is a reasonably sizeable number of bacterial *chassis* available for specific applications that have been developed (and continue to be refined) over the years, further developments are needed as a decisive step towards the adoption of a universal *chassis*. Having discussed the main phenotypic properties and metabolic characteristics of a handful of bacterial species that serve as functional *chassis* for bioproduction, in this section we present the challenges that we perceive as a significant hurdle to overcome in the near future – and we propose that tackling them should be an integral part of the whole process that starts from selecting a promising bacterial species until the establishment of a given bioprocess at the industrial scale (Fig. 4). Importantly, these issues are known to be important for several bacterial *chassis*, even when the phenomena described below have been mostly described in *E*. coli‐derived *chassis*.

Unsolved aspects of microbial metabolism still pose considerable challenges when selecting a given bacterial *chassis* for executing specific (biochemical) functions. One of these problems is the *loss of catalytic efficiency* when the production process is scaled‐up in large bioreactors, compatible with industrial fermentations – which serves as a reminder that metabolic efficiency at the laboratory scale hardly means high fitness in an industrial bioreactor. As recently shown for a recombinant *E*. *coli* strain carrying genes needed for MVA synthesis cultured in a rich medium, cells engineered for bioproduction usually lose the implanted catalytic functions in several generations due to an evolutionary process in which enhanced fitness is selected for, which usually implies blocking or elimination of heterologous pathways (Rugbjerg *et al*., 2018). One of the main reasons behind this general effect is the presence of heterogeneities in the metabolism across the entire cell population (Nikel *et al*., 2015b), that could be either *endogenous* (i.e. arising from the native genomic and metabolic properties of the cells) or *exogenous* (i.e. due to the burden that producing a chemical compound at high yields usually imposes on the engineered cells) (Akkaya *et al*., 2018). Tackling this problem is therefore one of the major challenges in bringing bacterial *chassis* to actual industrial‐scale bioproduction. In connection with this aspect, the evolution of alternative metabolic architectures in the engineered biochemical network (as opposed to changes operated merely at the genetic level) during bioproduction will help designing more efficient bioprocesses (Nikel and de Lorenzo, 2018).

*Metabolic orthogonality* is a major objective (as well as a significant challenge) in the field of metabolic engineering. The design of authentic orthogonal metabolic pathways, a concept referring to the minimization (and, ideally, suppression) of potential interactions between the synthetic metabolic pathway to be implanted and the extant metabolism of the bacterial *chassis*, has become a holy grail to optimize bioproduction (Pandit *et al*., 2017). A much needed further step into this direction would be to rationally combine the different tools so far developed for genome engineering and experimentally curated computational prediction [e.g. *retrosynthesis approaches* (Delépine *et al*., 2018)] for designing entirely novel (i.e. synthetic) metabolic architectures that can be plugged‐in into any given bacterial *chassis*. The emerging field of designing and implementing orthogonal metabolisms is being boosted by applying sophisticated strategies for enzyme engineering and *de novo* protein design (Erb *et al*., 2017; Arnold, 2018). Such tailor‐made enzymes, potentially able to catalyze novel, non‐natural biochemical reactions, hold the promise of paving the way for the design of *portable* synthetic metabolisms that can be integrated in any given *chassis*. Automation and standardization, two core tenets of synthetic biology, should be incorporated in the workflow of designing orthogonal metabolisms as well. The combination of these approaches would result in the efficient design of *trans*‐metabolisms for bioproduction of virtually any chemical structure in a bacterial *chassis* of choice.

In close connection with engineering completely novel *trans*‐metabolisms, the field of metabolic engineering is currently primed for the expansion of products in the biotechnological agenda – as industrial‐scale bioproduction is still largely limited to a handful of chemicals that exist only naturally in biological systems. Accessing new‐to‐Nature products through metabolic engineering is not only desirable but an actual necessity in a rapidly changing world in which access to natural, fossil‐based resources is becoming critical. *Redesigning the biochemical palate* of bacterial *chassis* is the way forward to access these novel products, and rewriting the metabolic blueprint to broaden the chemical repertoire achievable by engineered cells is a condition to access novel products (Hossain *et al*., 2018). Note that such ambitious objective calls for deepening our current understanding of the very core metabolic wiring in bacteria, a subject that has recently gained much attention after quite some time of (undeserved) ostracism (Aslan *et al*., 2017). Including rare chemical elements into the biochemical agenda of bacterial *chassis*, for example halogens and boron (Kan *et al*., 2017), will revolutionize bioproduction by multiplying the catalytic power of bacterial cell factories. Yet again, these operations would be enabled by an in‐depth knowledge of microbial metabolism, and standardized workflows should be developed to access these novel biochemistries.

Finally, and in addition to all these scientific and technical aspects, legislative issues will play a decisive role in the practical implementation of novel bacterial *chassis* for bioproduction. The development of suitable regulatory frameworks will be needed to bring superior biocatalysts into the biotechnological arena by tackling and overcoming legal restrictions. In any case, and irrespective of the current hurdles, the whole field of metabolic engineering is witnessing an exceptional increase in the number of bacterial *chassis* adopted for bioproduction, especially considering that the revolution brought about by the implementation of sophisticated tools for genome engineering (e.g. CRISPR/Cas9‐based approaches) has eliminated many of the common barriers that used to hinder the *à la carte* construction of bacterial *chassis*. The time has definitely come to capitalize on the knowledge amassed over the years for the smart design and construction of robust bacterial cell factories by combining *in silico* and wet‐laboratory approaches.

## Conflict of interest

None declared.

## References

[mbt213292-bib-0001] Acevedo‐Rocha, C.G. , Fang, G. , Schmidt, M. , Ussery, D.W. , and Danchin, A. (2013) From essential to persistent genes: a functional approach to constructing synthetic life. Trends Genet 29: 273–279. 23219343 10.1016/j.tig.2012.11.001 PMC3642372

[mbt213292-bib-0002] Ajikumar, P.K. , Xiao, W.H. , Tyo, K.E.J. , Wang, Y. , Simeon, F. , Leonard, E. , *et al* (2010) Isoprenoid pathway optimization for taxol precursor overproduction in *Escherichia coli* . Science 330: 70–74. 20929806 10.1126/science.1191652 PMC3034138

[mbt213292-bib-0003] Akkaya, Ö. , Pérez‐Pantoja, D. , Calles, B. , Nikel, P.I. and de Lorenzo, V. (2018) The metabolic redox regime of *Pseudomonas putida* tunes its evolvability towards novel xenobiotic substrates. bioRxiv, In press 10.1101/314989 PMC6113623 30154264

[mbt213292-bib-0004] Anjum, A. , Zuber, M. , Zia, K.M. , Noreen, A. , Anjum, M.N. , and Tabasum, S. (2016) Microbial production of polyhydroxyalkanoates (PHAs) and its copolymers: a review of recent advancements. Int J Biol Macromol 89: 161–174. 27126172 10.1016/j.ijbiomac.2016.04.069

[mbt213292-bib-0005] Ara, K. , Ozaki, K. , Nakamura, K. , Yamane, K. , Sekiguchi, J. , and Ogasawara, N. (2007) *Bacillus minimum* genome factory: effective utilization of microbial genome information. Biotechnol Appl Biochem 46: 169–178. 17115975 10.1042/BA20060111

[mbt213292-bib-0006] Arnold, F.H. (2018) Directed evolution: bringing new chemistry to life. Angew Chem Int Ed Engl 57: 4143–4148. 29064156 10.1002/anie.201708408 PMC5901037

[mbt213292-bib-0007] Asahara, T. , and Mori, Y. (2010) Accumulation of gene‐targeted *Bacillus subtilis* mutations that enhance fermentative inosine production. Appl Microbiol Biotechnol 87: 2195–2207. 20524113 10.1007/s00253-010-2646-8

[mbt213292-bib-0008] Aslan, S. , Noor, E. , and Bar‐Even, A. (2017) Holistic bioengineering: rewiring central metabolism for enhanced bioproduction. Biochem J 474: 3935–3950. 29146872 10.1042/BCJ20170377 PMC5688466

[mbt213292-bib-0009] Atsumi, S. , Wu, T.Y. , Machado, I.M.P. , Huang, W.C. , Chen, P.Y. , Pellegrini, M. , and Liao, J.C. (2010) Evolution, genomic analysis, and reconstruction of isobutanol tolerance in *Escherichia coli* . Mol Syst Biol 6: 449. 21179021 10.1038/msb.2010.98 PMC3018172

[mbt213292-bib-0010] Baba, T. , Ara, T. , Hasegawa, M. , Takai, Y. , Okumura, Y. , Baba, M. , *et al* (2006) Construction of *Escherichia coli* K‐12 in‐frame, single‐gene knockout mutants: the Keio collection. Mol Syst Biol 2: 2006.0008. 10.1038/msb4100050 PMC1681482 16738554

[mbt213292-bib-0011] Baeshen, M.N. , Al‐Hejin, A.M. , Bora, R.S. , Ahmed, M.M.M. , Ramadan, H.A.I. , Saini, K.S. , *et al* (2015) Production of biopharmaceuticals in *E. coli*: current scenario and future perspectives. J Microbiol Biotechnol 25: 953–962. 25737124 10.4014/jmb.1412.12079

[mbt213292-bib-0012] Bajaj, I. , and Singhal, R. (2011) Poly(glutamic acid)—An emerging biopolymer of commercial interest. Bioresour Technol 102: 5551–5561. 21377358 10.1016/j.biortech.2011.02.047

[mbt213292-bib-0013] Becker, J. , and Wittmann, C. (2012) Bio‐based production of chemicals, materials and fuels—*Corynebacterium glutamicum* as versatile cell factory. Curr Opin Biotechnol 23: 631–640. 22138494 10.1016/j.copbio.2011.11.012

[mbt213292-bib-0014] Beckham, G.T. , Johnson, C.W. , Karp, E.M. , Salvachúa, D. , and Vardon, D.R. (2016) Opportunities and challenges in biological lignin valorization. Curr Opin Biotechnol 42: 40–53. 26974563 10.1016/j.copbio.2016.02.030

[mbt213292-bib-0015] van Beilen, J.B. , Holtackers, R. , Lüscher, D. , Bauer, U. , Witholt, B. , and Duetz, W.A. (2005) Biocatalytic production of perillyl alcohol from limonene by using a novel *Mycobacterium* sp. cytochrome P450 alkane hydroxylase expressed in *Pseudomonas putida* . Appl Environ Microbiol 71: 1737–1744. 15811996 10.1128/AEM.71.4.1737-1744.2005 PMC1082528

[mbt213292-bib-0016] Beites, T. , and Mendes, M.V. (2015) *Chassis* optimization as a cornerstone for the application of synthetic biology based strategies in microbial secondary metabolism. Front Microbiol 6: 906. 26441855 10.3389/fmicb.2015.00906 PMC4563238

[mbt213292-bib-0017] Belda, E. , van Heck, R.G.A. , López‐Sánchez, M.J. , Cruveiller, S. , Barbe, V. , Fraser, C. , *et al* (2016) The revisited genome of *Pseudomonas putida* KT2440 enlightens its value as a robust metabolic *chassis* . Environ Microbiol 18: 3403–3424. 26913973 10.1111/1462-2920.13230

[mbt213292-bib-0018] Beuttler, H. , Hoffmann, J. , Jeske, M. , Hauer, B. , Schmid, R.D. , Altenbuchner, J. , and Urlacher, V.B. (2011) Biosynthesis of zeaxanthin in recombinant *Pseudomonas putida* . Appl Microbiol Biotechnol 89: 1137–1147. 21038098 10.1007/s00253-010-2961-0

[mbt213292-bib-0019] Bidart, G.N. , Ruiz, J.A. , de Almeida, A. , Méndez, B.S. , and Nikel, P.I. (2012) Manipulation of the anoxic metabolism in *Escherichia coli* by ArcB deletion variants in the ArcBA two‐component system. Appl Environ Microbiol 78: 8784–8794. 23064346 10.1128/AEM.02558-12 PMC3502932

[mbt213292-bib-0020] Blank, L.M. , Ionidis, G. , Ebert, B.E. , Bühler, B. , and Schmid, A. (2008) Metabolic response of *Pseudomonas putida* during redox biocatalysis in the presence of a second octanol phase. FEBS J 275: 5173–5190. 18803670 10.1111/j.1742-4658.2008.06648.x

[mbt213292-bib-0021] Bloor, A.E. , and Cranenburgh, R.M. (2006) An efficient method of selectable marker gene excision by Xer recombination for gene replacement in bacterial chromosomes. Appl Environ Microbiol 72: 2520–2525. 16597952 10.1128/AEM.72.4.2520-2525.2006 PMC1449051

[mbt213292-bib-0022] Blount, Z.D. (2015) The unexhausted potential of *E*. *coli* . eLife 4, e05826. 10.7554/eLife.05826 PMC4373459 25807083

[mbt213292-bib-0023] Blount, Z.D. , Borland, C.Z. , and Lenski, R.E. (2008) Historical contingency and the evolution of a key innovation in an experimental population of *Escherichia coli* . Proc Nat Acad Sci USA 105: 7899–7906. 18524956 10.1073/pnas.0803151105 PMC2430337

[mbt213292-bib-0024] Bongers, M. , Chrysanthopoulos, P.K. , Behrendorff, J.B.Y.H. , Hodson, M.P. , Vickers, C.E. , and Nielsen, L.K. (2015) Systems analysis of methylerythritol‐phosphate pathway flux in *E*. *coli*: insights into the role of oxidative stress and the validity of lycopene as an isoprenoid reporter metabolite. Microb Cell Fact 14: 193. 26610700 10.1186/s12934-015-0381-7 PMC4662018

[mbt213292-bib-0025] Borg, Y. , Grigonyte, A.M. , Boeing, P. , Wolfenden, B. , Smith, P. , Beaufoy, W. , *et al* (2016) Open source approaches to establishing *Roseobacter* clade bacteria as synthetic biology *chassis* for biogeoengineering. PeerJ 4: e2031. 27441104 10.7717/peerj.2031 PMC4941783

[mbt213292-bib-0026] Brock, N.L. , Menke, M. , Klapschinski, T.A. , and Dickschat, J.S. (2014) Marine bacteria from the *Roseobacter* clade produce sulfur volatiles *via* amino acid and dimethylsulfoniopropionate catabolism. Org Biomol Chem 12: 4318–4323. 24848489 10.1039/c4ob00719k

[mbt213292-bib-0027] Burgard, A. , Burk, M.J. , Osterhout, R. , van Dien, S. , and Yim, H. (2016) Development of a commercial scale process for production of 1,4‐butanediol from sugar. Curr Opin Biotechnol 42: 118–125. 27132123 10.1016/j.copbio.2016.04.016

[mbt213292-bib-0028] Calero, P. , Jensen, S.I. , and Nielsen, A.T. (2016) Broad‐host‐range *ProUSER* vectors enable fast characterization of inducible promoters and optimization of *p*‐coumaric acid production in *Pseudomonas putida* KT2440. ACS Synth Biol 5: 741–753. 27092814 10.1021/acssynbio.6b00081

[mbt213292-bib-0029] Calero, P. , Jensen, S.I. , Bojanovič, K. , Lennen, R.M. , Koza, A. , and Nielsen, A.T. (2018) Genome‐wide identification of tolerance mechanisms toward *p*‐coumaric acid in *Pseudomonas putida* . Biotechnol Bioeng 115: 762–774. 29131301 10.1002/bit.26495 PMC5814926

[mbt213292-bib-0030] Carroll, A.L. , Case, A.E. , Zhang, A. and Atsumi, S. (2018) Metabolic engineering tools in model cyanobacteria. Metab Eng, In press 10.1016/j.ymben.2018.1003.1014 29588234

[mbt213292-bib-0031] del Castillo, T. , Ramos, J.L. , Rodríguez‐Herva, J.J. , Fuhrer, T. , Sauer, U. , and Duque, E. (2007) Convergent peripheral pathways catalyze initial glucose catabolism in *Pseudomonas putida*: genomic and flux analysis. J Bacteriol 189: 5142–5152. 17483213 10.1128/JB.00203-07 PMC1951859

[mbt213292-bib-0032] Chavarría, M. , Nikel, P.I. , Pérez‐Pantoja, D. , and de Lorenzo, V. (2013) The Entner‐Doudoroff pathway empowers *Pseudomonas putida* KT2440 with a high tolerance to oxidative stress. Environ Microbiol 15: 1772–1785. 23301697 10.1111/1462-2920.12069

[mbt213292-bib-0033] Chen, G.Q. , and Jiang, X.R. (2017) Engineering bacteria for enhanced polyhydroxyalkanoates (PHA) biosynthesis. Synth Syst Biotechnol 2: 192–197. 29318199 10.1016/j.synbio.2017.09.001 PMC5655382

[mbt213292-bib-0034] Chen, S. , Chu, J. , Zhuang, Y. , and Zhang, S. (2005) Enhancement of inosine production by *Bacillus subtilis* through suppression of carbon overflow by sodium citrate. Biotechnol Lett 27: 689–692. 16049735 10.1007/s10529-005-4686-1

[mbt213292-bib-0035] Chen, J. , Zhao, L. , Fu, G. , Zhou, W. , Sun, Y. , Zheng, P. and Sun, J. (2016) A novel strategy for protein production using non‑classical secretion pathway in *Bacillus subtilis* . Microb Cell Fact 15, 1–16. 27125780 10.1186/s12934-016-0469-8 PMC4850722

[mbt213292-bib-0036] Chen, R. , Zhu, L. , Lv, L. , Yao, S. , Li, B. , and Qian, J. (2017) Optimization of the extraction and purification of the compatible solute ectoine from *Halomonas elongate* in the laboratory experiment of a commercial production project. World J Microbiol Biotechnol 33: 1–7. 28488199 10.1007/s11274-017-2281-y

[mbt213292-bib-0037] Choe, D. , Cho, S. , Kim, S.C. , and Cho, B.K. (2016) Minimal genome: worthwhile or worthless efforts toward being smaller? Biotechnol J 11: 199–211. 26356135 10.1002/biot.201400838

[mbt213292-bib-0038] Commichau, F.M. , Alzinger, A. , Sande, R. , Bretzel, W. , Meyer, F.M. , Chevreux, B. , *et al* (2014) Overexpression of a non‐native deoxyxylulose‐dependent vitamin B6 pathway in *Bacillus subtilis* for the production of pyridoxine. Metab Eng 25: 38–49. 24972371 10.1016/j.ymben.2014.06.007

[mbt213292-bib-0039] Cotton, C.A. , Edlich‐Muth, C. , and Bar‐Even, A. (2018) Reinforcing carbon fixation: CO_2_ reduction replacing and supporting carboxylation. Curr Opin Biotechnol 49: 49–56. 28803187 10.1016/j.copbio.2017.07.014

[mbt213292-bib-0040] Dalia, T.N. , Hayes, C.A. , Stolyar, S. , Marx, C.J. , McKinlay, J.B. , and Dalia, A.B. (2017) Multiplex genome editing by natural transformation (*MuGENT*) for synthetic biology in *Vibrio natriegens* . ACS Synth Biol 6: 1650–1655. 28571309 10.1021/acssynbio.7b00116 PMC6519440

[mbt213292-bib-0041] Danchin, A. , and Fang, G. (2016) Unknown unknowns: essential genes in quest for function. Microb Biotechnol 9: 530–540. 27435445 10.1111/1751-7915.12384 PMC4993169

[mbt213292-bib-0042] Danchin, A. , and Sekowska, A. (2013) Constraints in the design of the synthetic bacterial *chassis* . Methods Microbiol 40: 39–67.

[mbt213292-bib-0043] Dantas, J.M. , Morgado, L. , Aklujkar, M. , Bruix, M. , Londer, Y.Y. , Schiffer, M. , *et al* (2015) Rational engineering of *Geobacter sulfurreducens* electron transfer components: a foundation for building improved *Geobacter*‐based bioelectrochemical technologies. Front Microbiol 6: 752. 26284042 10.3389/fmicb.2015.00752 PMC4519760

[mbt213292-bib-0044] Datsenko, K.A. , and Wanner, B.L. (2000) One‐step inactivation of chromosomal genes in *Escherichia coli* K‐12 using PCR products. Proc Natl Acad Sci USA 97: 6640–6645. 10829079 10.1073/pnas.120163297 PMC18686

[mbt213292-bib-0045] Davies, F.K. , Work, V.H. , Beliaev, A.S. , and Posewitz, M.C. (2014) Engineering limonene and bisabolene production in wild type and a glycogen‐deficient mutant of *Synechococcus* sp. PCC 7002. Front Bioeng Biotechnol 2: 21. 25152894 10.3389/fbioe.2014.00021 PMC4126464

[mbt213292-bib-0046] Davis, R. , Duane, G. , Kenny, S.T. , Cerrone, F. , Guzik, M.W. , Babu, R.P. , *et al* (2015) High cell density cultivation of *Pseudomonas putida* KT2440 using glucose without the need for oxygen enriched air supply. Biotechnol Bioeng 112: 725–733. 25311981 10.1002/bit.25474

[mbt213292-bib-0047] Delépine, B. , Duigou, T. , Carbonell, P. , and Faulon, J.L. (2018) *RetroPath2.0*: a retrosynthesis workflow for metabolic engineers. Metab Eng 45: 158–170. 29233745 10.1016/j.ymben.2017.12.002

[mbt213292-bib-0048] Dexter, J. , and Fu, P. (2009) Metabolic engineering of cyanobacteria for ethanol production. Energy Environ Sci 2: 857–864.

[mbt213292-bib-0049] van Dijl, J.M. , and Hecker, M. (2013) *Bacillus subtilis*: from soil bacterium to super‐secreting cell factory. Microb Cell Fact 12: 1–6. 23311580 10.1186/1475-2859-12-3 PMC3564730

[mbt213292-bib-0050] Domínguez‐Cuevas, P. , González‐Pastor, J.E. , Marqués, S. , Ramos, J.L. , and de Lorenzo, V. (2006) Transcriptional tradeoff between metabolic and stress‐response programs in *Pseudomonas putida* KT2440 cells exposed to toluene. J Biol Chem 281: 11981–11991. 16495222 10.1074/jbc.M509848200

[mbt213292-bib-0051] Doudna, J.A. , and Charpentier, E. (2014) The new frontier of genome engineering with CRISPR‐Cas9. Science 346: 1258096. 25430774 10.1126/science.1258096

[mbt213292-bib-0052] Dragosits, M. and Mattanovich, D. (2013) Adaptive laboratory evolution—Principles and applications for biotechnology. Microb Cell Fact 12, 64. 23815749 10.1186/1475-2859-12-64 PMC3716822

[mbt213292-bib-0053] Ducat, D.C. , Way, J.C. , and Silver, P.A. (2011) Engineering cyanobacteria to generate high‐value products. Trends Biotechnol 29: 95–103. 21211860 10.1016/j.tibtech.2010.12.003

[mbt213292-bib-0054] Duque, E. , Molina‐Henares, A.J. , de la Torre, J. , Molina‐Henares, M.A. , Castillo, T. , Lam, J. , and Ramos, J.L. (2007) Towards a genome‐wide mutant library of *Pseudomonas putida* strain KT2440 In Pseudomonas. Ramos J.L., and Filloux A. (eds). Dordrecht, The Netherlands: Springer, pp. 227–251.

[mbt213292-bib-0055] van Duuren, J.B. , Wijte, D. , Leprince, A. , Karge, B. , Puchałka, J. , Wery, J. , *et al* (2011) Generation of a *catR* deficient mutant of *P. putida* KT2440 that produces *cis, cis*‐muconate from benzoate at high rate and yield. J Biotechnol 156: 163–172. 21906639 10.1016/j.jbiotec.2011.08.030

[mbt213292-bib-0056] van Duuren, J.B. , Wijte, D. , Karge, B. , Martins dos Santos, V.A.P. , Yang, Y. , Mars, A.E. , and Eggink, G. (2012) pH‐stat fed‐batch process to enhance the production of *cis, cis*‐muconate from benzoate by *Pseudomonas putida* KT2440‐JD1. Biotechnol Prog 28: 85–92. 21954182 10.1002/btpr.709

[mbt213292-bib-0057] Dvořák, P. , Chrást, L. , Nikel, P.I. , Fedr, R. , Soucek, K. , Sedlacková, M. , *et al* (2015) Exacerbation of substrate toxicity by IPTG in *Escherichia coli* BL21(DE3) carrying a synthetic metabolic pathway. Microb Cell Fact 14: 201. 26691337 10.1186/s12934-015-0393-3 PMC4687329

[mbt213292-bib-0058] Dvořák, P. , Nikel, P.I. , Damborský, J. , and de Lorenzo, V. (2017) *Bioremediation 3.0*: engineering pollutant‐removing bacteria in the times of systemic biology. Biotechnol Adv 35: 845–866. 28789939 10.1016/j.biotechadv.2017.08.001

[mbt213292-bib-0059] Eagon, R.G. (1961) *Pseudomonas natriegens*, a marine bacterium with a generation time of less than 10 minutes. J Bacteriol 83: 736–737. 10.1128/jb.83.4.736-737.1962 PMC279347 13888946

[mbt213292-bib-0060] Endy, D. (2005) Foundations for engineering biology. Nature 438: 449–453. 16306983 10.1038/nature04342

[mbt213292-bib-0061] Erb, T.J. , Jones, P.R. , and Bar‐Even, A. (2017) Synthetic metabolism: metabolic engineering meets enzyme design. Curr Opin Chem Biol 37: 56–62. 28152442 10.1016/j.cbpa.2016.12.023 PMC7610756

[mbt213292-bib-0062] Faizal, I. , Dozen, K. , Hong, C.S. , Kuroda, A. , Takiguchi, N. , Ohtake, H. , *et al* (2005) Isolation and characterization of solvent‐tolerant *Pseudomonas putida* strain T‐57, and its application to biotransformation of toluene to cresol in a two‐phase (organic‐aqueous) system. J Ind Microbiol Biotechnol 32: 542–547. 15947959 10.1007/s10295-005-0253-y

[mbt213292-bib-0063] Feng, Y. , Liu, S. , Jiao, Y. , Gao, H. , Wang, M. , and Du, G. (2017) Enhanced extracellular production of L‐asparaginase from *Bacillus subtilis* 168 by *B. subtilis* WB600 through a combined strategy. Appl Microbiol Biotechnol 101: 1509–1520. 27796436 10.1007/s00253-016-7816-x

[mbt213292-bib-0064] Fernández‐Cabezón, L. , Galán, B. , and García, J.L. (2018) New insights on steroid biotechnology. Front Microbiol 9: 958. 29867863 10.3389/fmicb.2018.00958 PMC5962712

[mbt213292-bib-0065] Fredrickson, J.K. , Romine, M.F. , Beliaev, A.S. , Auchtung, J.M. , Driscoll, M.E. , Gardner, T.S. , *et al* (2008) Towards environmental systems biology of *Shewanella* . Nat Rev Microbiol 6: 592–603. 18604222 10.1038/nrmicro1947

[mbt213292-bib-0066] Fu, X.Z. , Tan, D. , Aibaidula, G. , Wu, Q. , Chen, J.C. , and Chen, G.Q. (2014) Development of *Halomonas* TD01 as a host for open production of chemicals. Metab Eng 23: 78–91. 24566041 10.1016/j.ymben.2014.02.006

[mbt213292-bib-0067] Fu, J. , Huo, G. , Feng, L. , Mao, Y. , Wang, Z. , Ma, H. , and Chen, T. (2016) Metabolic engineering of *Bacillus subtilis* for chiral pure *meso*‐2,3‐butanediol production. Biotechnol Biofuels 9: 90. 27099629 10.1186/s13068-016-0502-5 PMC4837526

[mbt213292-bib-0068] Gatenby, A.A. , Haynie, S.L. , Nagarajan, V. , Nair, R.V. , Nakamura, C.E. , Payne, M.S. , *et al* (1998) Method for the production of 1,3‐propanediol by recombinant organisms. Patent PCT/US1997/020292.

[mbt213292-bib-0069] Gerber, E. , Bernard, R. , Castang, S. , Chabot, N. , Coze, F. , Dreux‐Zigha, A. , *et al* (2015) *Deinococcus* as new *chassis* for industrial biotechnology: biology, physiology and tools. J Appl Microbiol 119: 1–10. 25809882 10.1111/jam.12808 PMC4682472

[mbt213292-bib-0070] Gibson, D.G. , Glass, J.I. , Lartigue, C. , Noskov, V.N. , Chuang, R.Y. , Algire, M.A. , *et al* (2010) Creation of a bacterial cell controlled by a chemically synthesized genome. Science 329: 52–57. 20488990 10.1126/science.1190719

[mbt213292-bib-0071] Gomez, J.G.C. , Méndez, B.S. , Nikel, P.I. , Pettinari, M.J. , Prieto, M.A. , and Silva, L.F. (2012) Making green polymers even greener: Towards sustainable production of polyhydroxyalkanoates from agroindustrial by‐products In Advances in applied biotechnology. Petre M. (ed). InTech: Rijeka, Croatia, pp. 41–62.

[mbt213292-bib-0072] Goodarzi, H. , Bennett, B.D. , Amini, S. , Reaves, M.L. , Hottes, A.K. , Rabinowitz, J.D. , and Tavazoie, S. (2010) Regulatory and metabolic rewiring during laboratory evolution of ethanol tolerance in *E*. *coli* . Mol Syst Biol 6: 378. 20531407 10.1038/msb.2010.33 PMC2913397

[mbt213292-bib-0073] Graf, N. , and Altenbuchner, J. (2014) Genetic engineering of *Pseudomonas putida* KT2440 for rapid and high‐yield production of vanillin from ferulic acid. Appl Microbiol Biotechnol 98: 137–149. 24136472 10.1007/s00253-013-5303-1

[mbt213292-bib-0074] Gu, Y. , Xu, X. , Wu, Y. , Niu, T. , Liu, Y. , Li, J. , *et al* (2018) Advances and prospects of *Bacillus subtilis* cellular factories: From rational design to industrial applications. Metab Eng, In press 10.1016/j.ymben.2018.1005.1006 29775652

[mbt213292-bib-0075] Halfmann, C. , Gu, L. , Gibbons, W. , and Zhou, R. (2014) Genetically engineering cyanobacteria to convert CO_2_, water, and light into the long‐chain hydrocarbon farnesene. Appl Microbiol Biotechnol 98: 9869–9877. 25301585 10.1007/s00253-014-6118-4

[mbt213292-bib-0076] Hashimoto, M. , Ichimura, T. , Mizoguchi, H. , Tanaka, K. , Fujimitsu, K. , Keyamura, K. , *et al* (2005) Cell size and nucleoid organization of engineered *Escherichia coli* cells with a reduced genome. Mol Microbiol 55: 137–149. 15612923 10.1111/j.1365-2958.2004.04386.x

[mbt213292-bib-0077] Heider, S.A. , and Wendisch, V.F. (2015) Engineering microbial cell factories: metabolic engineering of *Corynebacterium glutamicum* with a focus on non‐natural products. Biotechnol J 10: 1170–1184. 26216246 10.1002/biot.201400590

[mbt213292-bib-0078] Herring, C.D. , Raghunathan, A. , Honisch, C. , Patel, T. , Applebee, M.K. , Joyce, A.R. , *et al* (2006) Comparative genome sequencing of *Escherichia coli* allows observation of bacterial evolution on a laboratory timescale. Nat Genet 38: 1406–1412. 17086184 10.1038/ng1906

[mbt213292-bib-0079] Hiltner, J.K. , Hunter, I.S. , and Hoskisson, P.A. (2015) Tailoring specialized metabolite production in *Streptomyces* . Adv Appl Microbiol 91: 237–255. 25911235 10.1016/bs.aambs.2015.02.002

[mbt213292-bib-0080] Hirokawa, Y. , Kawano, H. , Tanaka‐Masuda, K. , Nakamura, N. , Nakagawa, A. , Ito, M. , *et al* (2013) Genetic manipulations restored the growth fitness of reduced‐genome *Escherichia coli* . J Biosci Bioeng 116: 52–58. 23477741 10.1016/j.jbiosc.2013.01.010

[mbt213292-bib-0081] Hirokawa, Y. , Maki, Y. , and Hanai, T. (2017) Improvement of 1,3‐propanediol production using an engineered cyanobacterium, *Synechococcus elongatus* by optimization of the gene expression level of a synthetic metabolic pathway and production conditions. Metab Eng 39: 192–199. 27998670 10.1016/j.ymben.2016.12.001

[mbt213292-bib-0082] Hoffart, E. , Grenz, S. , Lange, J. , Nitschel, R. , Müller, F. , Schwentner, A. , *et al* (2017) High substrate uptake rates empower *Vibrio natriegens* as production host for industrial biotechnology. Appl Environ Microbiol 83, e01614–e01617. 10.1128/AEM.01614-17 PMC5666143 28887417

[mbt213292-bib-0083] Hoffmann, N. , and Rehm, B.H.A. (2004) Regulation of polyhydroxyalkanoate biosynthesis in *Pseudomonas putida* and *Pseudomonas aeruginosa* . FEMS Microbiol Lett 237: 1–7. 15268931 10.1016/j.femsle.2004.06.029

[mbt213292-bib-0084] Hohmann, H.P. , van Dijl, J.M. , Krishnappa, L. and Prágai, Z. (2017) Host organisms: *Bacillus subtilis* In Industrial Biotechnology: Microorganisms. Wittmann C. and Liao J.C. (eds.). Weinheim, Germany: Wiley‐VCH Verlag GmbH & Co., pp. 221–297.

[mbt213292-bib-0085] Hossain, G.S. , Nadarajan, S.P. , Zhang, L. , Ng, T.K. , Foo, J.L. , Ling, H. , *et al* (2018) Rewriting the metabolic blueprint: advances in pathway diversification in microorganisms. Front Microbiol 9: 155. 29483901 10.3389/fmicb.2018.00155 PMC5816047

[mbt213292-bib-0086] Huang, H.T. (1961) Production of L‐threonine by auxotrophic mutants of *Escherichia coli* . Appl Microbiol 9: 419–424. 13716394 10.1128/am.9.5.419-424.1961 PMC1057759

[mbt213292-bib-0087] Hüsken, L.E. , Dalm, M.C.F. , Tramper, J. , Wery, J. , de Bont, J.A.M. , and Beeftink, R. (2001) Integrated bioproduction and extraction of 3‐methylcatechol. J Biotechnol 88: 11–19. 11377761 10.1016/s0168-1656(01)00252-8

[mbt213292-bib-0088] Hutchison, C.A. , Chuang, R.Y. , Noskov, V.N. , Assad‐García, N. , Deerinck, T.J. , Ellisman, M.H. , *et al* (2016) Design and synthesis of a minimal bacterial genome. Science 351, aad6253. 27013737 10.1126/science.aad6253

[mbt213292-bib-0089] Inoue, A. , and Horikoshi, K. (1989) A *Pseudomonas* thrives in high concentrations of toluene. Nature 338: 264–266.

[mbt213292-bib-0090] Jang, Y.S. , Lee, J.Y. , Lee, J. , Park, J.H. , Im, J.A. , Eom, M.H. , *et al* (2012) Enhanced butanol production obtained by reinforcing the direct butanol‐forming route in *Clostridium acetobutylicum* . MBio 3, e00314–12. 23093384 10.1128/mBio.00314-12 PMC3482502

[mbt213292-bib-0091] Jiang, W. , Bikard, D. , Cox, D. , Zhang, F. , and Marraffini, L.A. (2013) RNA‐guided editing of bacterial genomes using CRISPR‐Cas systems. Nat Biotechnol 31: 233–239. 23360965 10.1038/nbt.2508 PMC3748948

[mbt213292-bib-0092] Jiang, Y. , Chen, B. , Duan, C. , Sun, B. , Yang, J. , and Yang, S. (2015) Multigene editing in the *Escherichia coli* genome *via* the CRISPR‐Cas9 system. Appl Environ Microbiol 81: 2506–2514. 25636838 10.1128/AEM.04023-14 PMC4357945

[mbt213292-bib-0093] Jiménez, J.I. , Miñambres, B. , García, J.L. , and Díaz, E. (2002) Genomic analysis of the aromatic catabolic pathways from *Pseudomonas putida* KT2440. Environ Microbiol 4: 824–841. 12534466 10.1046/j.1462-2920.2002.00370.x

[mbt213292-bib-0094] Jin, P. , Kang, Z. , Yuan, P. , Du, G. , and Chen, J. (2016) Production of specific‐molecular‐weight hyaluronan by metabolically engineered *Bacillus subtilis* 168. Metab Eng 35: 21–30. 26851304 10.1016/j.ymben.2016.01.008

[mbt213292-bib-0095] Jullesson, D. , David, F. , Pfleger, B. , and Nielsen, J. (2015) Impact of synthetic biology and metabolic engineering on industrial production of fine chemicals. Biotechnol Adv 33: 1395–1402. 25728067 10.1016/j.biotechadv.2015.02.011

[mbt213292-bib-0096] Kaczmarzyk, D. , Cengic, I. , Yao, L. , and Hudson, E.P. (2018) Diversion of the long‐chain acyl‐ACP pool in *Synechocystis* to fatty alcohols through CRISPRi repression of the essential phosphate acyltransferase PlsX. Metab Eng 45: 59–66. 29199103 10.1016/j.ymben.2017.11.014

[mbt213292-bib-0097] Kan, S.B.J. , Huang, X. , Gumulya, Y. , Chen, K. , and Arnold, F.H. (2017) Genetically programmed chiral organoborane synthesis. Nature 552: 132–136. 29186119 10.1038/nature24996 PMC5819735

[mbt213292-bib-0098] Kaneko, T. , Tanaka, A. , Sato, S. , Kotani, H. , Sazuka, T. , Miyajima, N. , *et al* (1995) Sequence analysis of the genome of the unicellular cyanobacterium *Synechocystis* sp. strain PCC6803. I. Sequence features in the 1 Mb region from map positions 64% to 92% of the genome. DNA Res 2: 153–166. 8590279 10.1093/dnares/2.4.153

[mbt213292-bib-0099] Keasling, J.D. (2010) Manufacturing molecules through metabolic engineering. Science 330: 1355–1358. 21127247 10.1126/science.1193990

[mbt213292-bib-0100] Kiener, A. (1992) Enzymatic oxidations of methyl groups on aromatic heterocycles: a versatile method for the preparation of heteroaromatics carboxylic acids. Angew Chem Int Ed Eng 6: 774–775.

[mbt213292-bib-0101] Kim, S.W. , and Keasling, J.D. (2001) Metabolic engineering of the nonmevalonate isopentenyl diphosphate synthesis pathway in *Escherichia coli* enhances lycopene production. Biotechnol Bioeng 72: 408–415. 11180061 10.1002/1097-0290(20000220)72:4<408::aid-bit1003>3.0.co;2-h

[mbt213292-bib-0102] Kim, J. , Oliveros, J.C. , Nikel, P.I. , de Lorenzo, V. , and Silva‐Rocha, R. (2013) Transcriptomic fingerprinting of *Pseudomonas putida* under alternative physiological regimes. Environ Microbiol Rep 5: 883–891. 24249296 10.1111/1758-2229.12090

[mbt213292-bib-0103] Komatsu, M. , Uchiyama, T. , Omura, S. , Cane, D.E. , and Ikeda, H. (2010) Genome‐minimized *Streptomyces* host for the heterologous expression of secondary metabolism. Proc Natl Acad Sci USA 107: 2646–2651. 20133795 10.1073/pnas.0914833107 PMC2823899

[mbt213292-bib-0104] Komatsu, M. , Komatsu, K. , Koiwai, H. , Yamada, Y. , Kozone, I. , Izumikawa, M. , *et al* (2013) Engineered *Streptomyces avermitilis* host for heterologous expression of biosynthetic gene cluster for secondary metabolites. ACS Synth Biol 2: 384–396. 23654282 10.1021/sb3001003 PMC3932656

[mbt213292-bib-0105] Koo, B.M. , Kritikos, G. , Farelli, J.D. , Todor, H. , Tong, K. , Kimsey, H. , *et al* (2017) Construction and analysis of two genome‐scale deletion libraries for *Bacillus subtilis* . Cell Syst 4, 291–305.e297 28189581 10.1016/j.cels.2016.12.013 PMC5400513

[mbt213292-bib-0106] Kosa, M. , and Ragauskas, A.J. (2013) Lignin to lipid bioconversion by oleaginous *Rhodococci* . Green Chem 15: 2070–2074.

[mbt213292-bib-0107] Kuepper, J. , Dickler, J. , Biggel, M. , Behnken, S. , Jäger, G. , Wierckx, N. , and Blank, L.M. (2015) Metabolic engineering of *Pseudomonas putida* KT2440 to produce anthranilate from glucose. Front Microbiol 6: 1–9. 26635771 10.3389/fmicb.2015.01310 PMC4656820

[mbt213292-bib-0108] Kumar, V. , and Park, S. (2018) Potential and limitations of *Klebsiella pneumoniae* as a microbial cell factory utilizing glycerol as the carbon source. Biotechnol Adv 36: 150–167. 29056473 10.1016/j.biotechadv.2017.10.004

[mbt213292-bib-0109] Kunst, F. , and Rapoport, G. (1995) Salt stress is an environmental signal affecting degradative enzyme synthesis in *Bacillus subtilis* . J Bacteriol 177: 2403–2407. 7730271 10.1128/jb.177.9.2403-2407.1995 PMC176898

[mbt213292-bib-0110] Kunst, F. , Ogasawara, N. , Moszer, I. , Albertini, A.M. , Alloni, G. , Azevedo, V. , *et al* (1997) The complete genome sequence of the Gram‐positive bacterium *Bacillus subtilis* . Nature 390: 249–256. 9384377 10.1038/36786

[mbt213292-bib-0111] Kusakabe, T. , Tatsuke, T. , Tsuruno, K. , and Hirokawa, Y. (2013) Engineering a synthetic pathway in cyanobacteria for isopropanol production directly from carbon dioxide and light. Metab Eng 20: 101–108. 24076145 10.1016/j.ymben.2013.09.007

[mbt213292-bib-0112] Kushwaha, M. , Rostain, W. , Prakash, S. , Duncan, J.N. , and Jaramillo, A. (2016) Using RNA as molecular code for programming cellular function. ACS Synth Biol 5: 795–809. 26999422 10.1021/acssynbio.5b00297

[mbt213292-bib-0113] Kusumawardhani, H. , Hosseini, R. and de Winde, J.H. (2018) Solvent tolerance in bacteria: fulfilling the promise of the Biotech Era? Trends Biotechnol, In press 10.1016/j.tibtech.2018.1004.1007 29778531

[mbt213292-bib-0114] LaCroix, R.A. , Sandberg, T.E. , O'Brien, E.J. , Utrilla, J. , Ebrahim, A. , Guzmán, G.I. , *et al* (2015) Use of adaptive laboratory evolution to discover key mutations enabling rapid growth of *Escherichia coli* K‐12 MG1655 on glucose minimal medium. Appl Environ Microbiol 81: 17–30. 25304508 10.1128/AEM.02246-14 PMC4272732

[mbt213292-bib-0115] Lai, B. , Yu, S. , Bernhardt, P.V. , Rabaey, K. , Virdis, B. , and Krömer, J.O. (2016) Anoxic metabolism and biochemical production in *Pseudomonas putida* F1 driven by a bioelectrochemical system. Biotechnol Biofuels 9: 39. 26893611 10.1186/s13068-016-0452-y PMC4758010

[mbt213292-bib-0116] Lan, E.I. , and Liao, J.C. (2011) Metabolic engineering of cyanobacteria for 1‐butanol production from carbon dioxide. Metab Eng 13: 353–363. 21569861 10.1016/j.ymben.2011.04.004

[mbt213292-bib-0117] Lee, J.H. , and Wendisch, V.F. (2017) Biotechnological production of aromatic compounds of the extended shikimate pathway from renewable biomass. J Biotechnol 257: 211–221. 27871872 10.1016/j.jbiotec.2016.11.016

[mbt213292-bib-0118] Lee, H.H. , Ostrov, N. , Wong, B.G. , Gold, M.A. , Khalil, A.S. and Church, G.M. (2016) *Vibrio natriegens*, a new genomic powerhouse. bioRxiv, In press 10.1101/058487

[mbt213292-bib-0119] Leprince, A. , de Lorenzo, V. , Völler, P. , van Passel, M.W.J. , and Martins dos Santos, V.A.P. (2012) Random and cyclical deletion of large DNA segments in the genome of *Pseudomonas putida* . Environ Microbiol 14: 1444–1453. 22429517 10.1111/j.1462-2920.2012.02730.x PMC3429869

[mbt213292-bib-0120] Li, R. , and Townsend, C.A. (2006) Rational strain improvement for enhanced clavulanic acid production by genetic engineering of the glycolytic pathway in *Streptomyces clavuligerus* . Metab Eng 8: 240–252. 16530442 10.1016/j.ymben.2006.01.003

[mbt213292-bib-0121] Lieder, S. , Nikel, P.I. , de Lorenzo, V. , and Takors, R. (2015) Genome reduction boosts heterologous gene expression in *Pseudomonas putida* . Microb Cell Fact 14: 23. 25890048 10.1186/s12934-015-0207-7 PMC4352270

[mbt213292-bib-0122] Lin, C.C. , Joe, C. , Yap, S. , Kan, S.C. , Hsueh, N.C. , Yang, L.Y. , *et al* (2017) Deciphering characteristics of the designer cellulosome from *Bacillus subtilis* WB800N *via* enzymatic analysis. Biochem Eng J 117: 147–155.

[mbt213292-bib-0123] Lindberg, P. , Park, S. , and Melis, A. (2010) Engineering a platform for photosynthetic isoprene production in cyanobacteria, using *Synechocystis* as the model organism. Metab Eng 12: 70–79. 19833224 10.1016/j.ymben.2009.10.001

[mbt213292-bib-0124] Linger, J.G. , Vardon, D.R. , Guarnieri, M.T. , Karp, E.M. , and Hunsinger, G.B. (2014) Lignin valorization through integrated biological funneling and chemical catalysis. Proc Nat Acad Sci USA 111: 12013–12018. 25092344 10.1073/pnas.1410657111 PMC4143016

[mbt213292-bib-0125] Liu, H. , and Deutschbauer, A.M. (2018) Rapidly moving new bacteria to model‐organism status. Curr Opin Biotechnol 51: 116–122. 29316481 10.1016/j.copbio.2017.12.006

[mbt213292-bib-0126] Liu, D. , Evans, T. , and Zhang, F. (2015) Applications and advances of metabolite biosensors for metabolic engineering. Metab Eng 31: 35–43. 26142692 10.1016/j.ymben.2015.06.008

[mbt213292-bib-0127] Liu, Y. , Li, J. , Du, G. , Chen, J. , and Liu, L. (2017a) Metabolic engineering of *Bacillus subtilis* fueled by systems biology: recent advances and future directions. Biotechnol Adv 35: 20–30. 27867004 10.1016/j.biotechadv.2016.11.003

[mbt213292-bib-0128] Liu, Y. , Zhu, Y. , Li, J. , Shin, H.D. , Chen, R.R. , Du, G. , *et al* (2017b) Modular pathway engineering of *Bacillus subtilis* for improved *N*‐acetylglucosamine production. Metab Eng 23: 42–52. 10.1016/j.ymben.2014.02.005 24560814

[mbt213292-bib-0129] Liu, Y. , Zhu, Y. , Ma, W. , Shin, H.D. , and Li, J. (2017c) Spatial modulation of key pathway enzymes by DNA‐guided scaffold system and respiration chain engineering for improved *N*‐acetylglucosamine production by *Bacillus subtilis* . Metab Eng 24: 61–69. 10.1016/j.ymben.2014.04.004 24815549

[mbt213292-bib-0130] Loeschcke, A. , and Thies, S. (2015) *Pseudomonas putida*—a versatile host for the production of natural products. Appl Microb Biotechnol 99: 6197–6214. 10.1007/s00253-015-6745-4 PMC4495716 26099332

[mbt213292-bib-0131] Loeschcke, A. , Markert, A. , Wilhelm, S. , Wirtz, A. , Rosenau, F. , Jaeger, K.E. , and Drepper, T. (2013) *TREX*: A universal tool for the transfer and expression of biosynthetic pathways in bacteria. ACS Synth Biol 2: 22–33. 23656323 10.1021/sb3000657

[mbt213292-bib-0132] Long, C.P. , González, J.E. , Cipolla, R.M. , and Antoniewicz, M.R. (2017) Metabolism of the fast‐growing bacterium *Vibrio natriegens* elucidated by ^13^C‐metabolic flux analysis. Metab Eng 44: 191–197. 29042298 10.1016/j.ymben.2017.10.008 PMC5845447

[mbt213292-bib-0133] de Lorenzo, V. , Sekowska, A. , and Danchin, A. (2015) Chemical reactivity drives spatiotemporal organisation of bacterial metabolism. FEMS Microbiol Rev 39: 96–119. 25227915 10.1111/1574-6976.12089

[mbt213292-bib-0134] Ma, S.M. , García, D.E. , Redding‐Johanson, A.M. , Friedland, G.D. , Chan, R. , Batth, T.S. , *et al* (2011) Optimization of a heterologous mevalonate pathway through the use of variant HMG‐CoA reductases. Metab Eng 13: 588–597. 21810477 10.1016/j.ymben.2011.07.001

[mbt213292-bib-0135] Maida, I. , Bosi, E. , Perrin, E. , Papaleo, M.C. , Orlandini, V. , Fondi, M. , *et al* (2013) Draft genome sequence of the fast‐growing bacterium *Vibrio natriegens* strain DSMZ 759. Genome Announc 1: e00648–13. 23969053 10.1128/genomeA.00648-13 PMC3751608

[mbt213292-bib-0136] Manabe, K. , Kageyama, Y. , Morimoto, T. , Shimizu, E. , Takahashi, H. , Kanaya, S. , *et al* (2013) Improved production of secreted heterologous enzyme in *Bacillus subtilis* strain MGB874 *via* modification of glutamate metabolism and growth conditions. Microb Cell Fact 12: 18. 23419162 10.1186/1475-2859-12-18 PMC3600796

[mbt213292-bib-0137] Marschall, L. , Sagmeister, P. , and Herwig, C. (2017) Tunable recombinant protein expression in *E. coli*: promoter systems and genetic constraints. Appl Microbiol Biotechnol 101: 501–512. 27999902 10.1007/s00253-016-8045-z PMC5566544

[mbt213292-bib-0138] Martens, T. , Gram, L. , Grossart, H.P. , Kessler, D. , Müller, R. , Simon, M. , *et al* (2007) Bacteria of the *Roseobacter* clade show potential for secondary metabolite production. Microb Ecol 54: 31–42. 17351813 10.1007/s00248-006-9165-2

[mbt213292-bib-0139] Martin, V.J.J. , Pitera, D.J. , Withers, S.T. , Newman, J.D. , and Keasling, J.D. (2003) Engineering a mevalonate pathway in *Escherichia coli* for production of terpenoids. Nat Biotechnol 21: 796–802. 12778056 10.1038/nbt833

[mbt213292-bib-0140] Martínez, J.A. , Bolívar, F. , and Escalante, A. (2015) Shikimic acid production in *Escherichia coli*: from classical metabolic engineering strategies to omics applied to improve its production. Front Bioeng Biotechnol 3: 145. 26442259 10.3389/fbioe.2015.00145 PMC4585142

[mbt213292-bib-0141] Martínez‐García, E. , and de Lorenzo, V. (2011) Engineering multiple genomic deletions in Gram‐negative bacteria: analysis of the multi‐resistant antibiotic profile of *Pseudomonas putida* KT2440. Environ Microbiol 13: 2702–2716. 21883790 10.1111/j.1462-2920.2011.02538.x

[mbt213292-bib-0142] Martínez‐García, E. , and de Lorenzo, V. (2016) The quest for the minimal bacterial genome. Curr Opin Biotechnol 42: 216–224. 27660908 10.1016/j.copbio.2016.09.001

[mbt213292-bib-0143] Martínez‐García, E. , and de Lorenzo, V. (2017) Molecular tools and emerging strategies for deep genetic/genomic refactoring of *Pseudomonas* . Curr Opin Biotechnol 47: 120–132. 28738232 10.1016/j.copbio.2017.06.013

[mbt213292-bib-0144] Martínez‐García, E. , Aparicio, T. , de Lorenzo, V. , and Nikel, P.I. (2014a) New transposon tools tailored for metabolic engineering of Gram‐negative microbial cell factories. Front Bioeng Biotechnol 2: 46. 25389526 10.3389/fbioe.2014.00046 PMC4211546

[mbt213292-bib-0145] Martínez‐García, E. , Nikel, P.I. , Aparicio, T. , and de Lorenzo, V. (2014b) *Pseudomonas 2.0*: genetic upgrading of *P*. *putida* KT2440 as an enhanced host for heterologous gene expression. Microb Cell Fact 13: 159. 25384394 10.1186/s12934-014-0159-3 PMC4230525

[mbt213292-bib-0146] Martínez‐García, E. , Nikel, P.I. , Chavarría, M. , and de Lorenzo, V. (2014c) The metabolic cost of flagellar motion in *Pseudomonas putida* KT2440. Environ Microbiol 16: 291–303. 24148021 10.1111/1462-2920.12309

[mbt213292-bib-0147] Martínez‐García, E. , Aparicio, T. , Goñi‐Moreno, A. , Fraile, S. , and de Lorenzo, V. (2015) *SEVA 2.0*: an update of the Standard European Vector Architecture for de‐/re‐construction of bacterial functionalities. Nucleic Acids Res 43: D1183–D1189. 25392407 10.1093/nar/gku1114 PMC4383931

[mbt213292-bib-0148] Martins dos Santos, V.A.P. , Heim, S. , Moore, E.R.B. , Strätz, M. , and Timmis, K.N. (2004) Insights into the genomic basis of niche specificity of *Pseudomonas putida* KT2440. Environ Microbiol 6: 1264–1286. 15560824 10.1111/j.1462-2920.2004.00734.x

[mbt213292-bib-0149] Matsui, H. , Sato, K. , Enei, H. , and Takinami, K. (1982) 5′‐Nucleotidase activity in improved inosine‐producing mutants of *Bacillus subtilis* . Agricult Biol Chem 46: 2347–2352.

[mbt213292-bib-0150] Matsumoto, T. , Tanaka, T. , and Kondo, A. (2017) Engineering metabolic pathways in *Escherichia coli* for constructing a “microbial *chassis*” for biochemical production. Biores Technol 245: 1362–1368. 10.1016/j.biortech.2017.05.008 28522199

[mbt213292-bib-0151] McCloskey, D. , Palsson, B.Ø. , and Feist, A.M. (2013) Basic and applied uses of genome‐scale metabolic network reconstructions of *Escherichia coli* . Mol Syst Biol 9: 661. 23632383 10.1038/msb.2013.18 PMC3658273

[mbt213292-bib-0152] McLeod, M.P. , Warren, R.L. , Hsiao, W.W. , Araki, N. , Myhre, M. , Fernandes, C. , *et al* (2006) The complete genome of *Rhodococcus* sp. RHA1 provides insights into a catabolic powerhouse. Proc Natl Acad Sci USA 103: 15582–15587. 17030794 10.1073/pnas.0607048103 PMC1622865

[mbt213292-bib-0153] Mi, J. , Becher, D. , Lubuta, P. , Dany, S. , Tusch, K. , Schewe, H. , *et al* (2014) *De novo* production of the monoterpenoid geranic acid by metabolically engineered *Pseudomonas putida* . Microb Cell Fact 13: 170. 25471523 10.1186/s12934-014-0170-8 PMC4266966

[mbt213292-bib-0154] Miroux, B. , and Walker, J.E. (1996) Over‐production of proteins in *Escherichia coli*: mutant hosts that allow synthesis of some membrane proteins and globular proteins at high levels. J Mol Biol 260: 289–298. 8757792 10.1006/jmbi.1996.0399

[mbt213292-bib-0155] Miyano, M. , Tanaka, K. , Ishikawa, S. , Takenaka, S. , Arribas, A.M. , Meijer, W.J.J. , and Yoshida, K. (2018) Rapid conjugative mobilization of a 100 kb segment of *Bacillus subtilis* chromosomal DNA is mediated by a helper plasmid with no ability for self‐transfer. Microb Cell Fact 17: 13. 29374463 10.1186/s12934-017-0855-x PMC5787278

[mbt213292-bib-0156] Mizoguchi, H. , Mori, H. , and Fujio, T. (2007) *Escherichia coli* minimum genome factory. Biotechnol Appl Biochem 46: 157–167. 17300222 10.1042/BA20060107

[mbt213292-bib-0157] Mohamed, E.T. , Wang, S. , Lennen, R.M. , Herrgård, M.J. , Simmons, B.A. , Singer, S.W. , and Feist, A.M. (2017) Generation of a platform strain for ionic liquid tolerance using adaptive laboratory evolution. Microb Cell Fact 16: 204. 29145855 10.1186/s12934-017-0819-1 PMC5691611

[mbt213292-bib-0158] Morimoto, T. , Kadoya, R. , Endo, K. , Tohata, M. , Sawada, K. , Liu, S. , *et al* (2008) Enhanced recombinant protein productivity by genome reduction in *Bacillus subtilis* . DNA Res 15: 73–81. 18334513 10.1093/dnares/dsn002 PMC2650625

[mbt213292-bib-0159] Morrone, D. , Lowry, L. , Determan, M.K. , Hershey, D.M. , Xu, M. , and Peters, R.J. (2010) Increasing diterpene yield with a modular metabolic engineering system in *E*. *coli*: comparison of MEV and MEP isoprenoid precursor pathway engineering. Appl Microbiol Biotechnol 85: 1893–1906. 19777230 10.1007/s00253-009-2219-x PMC2811251

[mbt213292-bib-0160] Mundhada, H. , Seoane, J.M. , Schneider, K. , Koza, A. , Christensen, H.B. , Klein, T. , *et al* (2017) Increased production of L‐serine in *Escherichia coli* through adaptive laboratory evolution. Metab Eng 39: 141–150. 27908688 10.1016/j.ymben.2016.11.008

[mbt213292-bib-0161] Nelson, K.E. , Weinel, C. , Paulsen, I.T. , Dodson, R.J. , Hilbert, H. , Martins dos Santos, V.A.P. , *et al* (2002) Complete genome sequence and comparative analysis of the metabolically versatile *Pseudomonas putida* KT2440. Environ Microbiol 4: 799–808. 12534463 10.1046/j.1462-2920.2002.00366.x

[mbt213292-bib-0162] Ni, Y. , and Sun, Z. (2009) Recent progress on industrial fermentative production of acetone‐butanol‐ethanol by *Clostridium acetobutylicum* in China. Appl Microbiol Biotechnol 83: 415–423. 19430776 10.1007/s00253-009-2003-y

[mbt213292-bib-0163] Nicolaou, S.A. , Gaida, S.M. , and Papoutsakis, E.T. (2010) A comparative view of metabolite and substrate stress and tolerance in microbial bioprocessing: from biofuels and chemicals, to biocatalysis and bioremediation. Metab Eng 12: 307–331. 20346409 10.1016/j.ymben.2010.03.004

[mbt213292-bib-0164] Nielsen, J. , and Keasling, J.D. (2016) Engineering cellular metabolism. Cell 164: 1185–1197. 26967285 10.1016/j.cell.2016.02.004

[mbt213292-bib-0165] Nijkamp, K. , van Luijk, N. , de Bont, J.A.M. , and Wery, J. (2005) The solvent‐tolerant *Pseudomonas putida* S12 as host for the production of cinnamic acid from glucose. Appl Microbiol Biotechnol 69: 170–177. 15824922 10.1007/s00253-005-1973-7

[mbt213292-bib-0166] Nikel, P.I. , and de Lorenzo, V. (2013) Engineering an anaerobic metabolic regime in *Pseudomonas putida* KT2440 for the anoxic biodegradation of 1,3‐dichloroprop‐1‐ene. Metab Eng 15: 98–112. 23149123 10.1016/j.ymben.2012.09.006

[mbt213292-bib-0167] Nikel, P.I. , and de Lorenzo, V. (2018) *Pseudomonas putida* as a functional *chassis* for industrial biocatalysis: from native biochemistry to *trans*‐metabolism. Metab Eng In press. 10.1016/j.ymben.2018.1005.1005. 29758287

[mbt213292-bib-0168] Nikel, P.I. , Kim, J. , and de Lorenzo, V. (2014a) Metabolic and regulatory rearrangements underlying glycerol metabolism in *Pseudomonas putida* KT2440. Environ Microbiol 16: 239–254. 23967821 10.1111/1462-2920.12224

[mbt213292-bib-0169] Nikel, P.I. , Martínez‐García, E. , and de Lorenzo, V. (2014b) Biotechnological domestication of pseudomonads using synthetic biology. Nat Rev Microbiol 12: 368–379. 24736795 10.1038/nrmicro3253

[mbt213292-bib-0170] Nikel, P.I. , Chavarría, M. , Fuhrer, T. , Sauer, U. , and de Lorenzo, V. (2015a) *Pseudomonas putida* KT2440 strain metabolizes glucose through a cycle formed by enzymes of the Entner‐Doudoroff, Embden‐Meyerhof‐Parnas, and pentose phosphate pathways. J Biol Chem 290: 25920–25932. 26350459 10.1074/jbc.M115.687749 PMC4646247

[mbt213292-bib-0171] Nikel, P.I. , Romero‐Campero, F.J. , Zeidman, J.A. , Goñi‐Moreno, A. and de Lorenzo, V. (2015b) The glycerol‐dependent metabolic persistence of *Pseudomonas putida* KT2440 reflects the regulatory logic of the GlpR repressor. mBio 6, e00340‐15. 25827416 10.1128/mBio.00340-15 PMC4453509

[mbt213292-bib-0172] Nikel, P.I. , Chavarría, M. , Danchin, A. , and de Lorenzo, V. (2016) From dirt to industrial applications: *Pseudomonas putida* as a synthetic biology *chassis* for hosting harsh biochemical reactions. Curr Opin Chem Biol 34: 20–29. 27239751 10.1016/j.cbpa.2016.05.011

[mbt213292-bib-0173] Noda, S. , Shirai, T. , Oyama, S. , and Kondo, A. (2016) Metabolic design of a platform *Escherichia coli* strain producing various chorismate derivatives. Metab Eng 33: 119–129. 26654797 10.1016/j.ymben.2015.11.007

[mbt213292-bib-0174] Nogales, J. , Palsson, B.Ø. , and Thiele, I. (2008) A genome‐scale metabolic reconstruction of *Pseudomonas putida* KT2440: *i*JN746 as a cell factory. BMC Syst Biol 2: 79. 18793442 10.1186/1752-0509-2-79 PMC2569920

[mbt213292-bib-0175] Nogales, J. , Gudmundsson, S. , Knight, E.M. , Palsson, B.Ø. , and Thiele, I. (2012) Detailing the optimality of photosynthesis in cyanobacteria through systems biology analysis. Proc Natl Acad Sci USA 109: 2678–2683. 22308420 10.1073/pnas.1117907109 PMC3289291

[mbt213292-bib-0176] Nogales, J. , Gudmundsson, S. , Duque, E. , Ramos, J.L. and Palsson, B.Ø. (2017) Expanding the computable reactome in *Pseudomonas putida* reveals metabolic cycles providing robustness. bioRxiv, In press. 10.1101/139121

[mbt213292-bib-0177] Notley‐McRobb, L. , and Ferenci, T. (1999) Adaptive *mgl*‐regulatory mutations and genetic diversity evolving in glucose‐limited *Escherichia coli* populations. Environ Microbiol 1: 33–43. 11207716 10.1046/j.1462-2920.1999.00002.x

[mbt213292-bib-0178] Nozzi, N.E. , Oliver, J.W.K. , and Atsumi, S. (2013) Cyanobacteria as a platform for biofuel production. Front Bioeng Biotechnol 1: 7. 25022311 10.3389/fbioe.2013.00007 PMC4090892

[mbt213292-bib-0179] O'Brien, E.J. , Monk, J.M. , and Palsson, B.Ø. (2015) Using genome‐scale models to predict biological capabilities. Cell 161: 971–987. 26000478 10.1016/j.cell.2015.05.019 PMC4451052

[mbt213292-bib-0180] Oh, Y.K. , Palsson, B.Ø. , Park, S.M. , Schilling, C.H. , and Mahadevan, R. (2007) Genome‐scale reconstruction of metabolic network in *Bacillus subtilis* based on high‐throughput phenotyping and gene essentiality data. J Biol Chem 282: 28791–28799. 17573341 10.1074/jbc.M703759200

[mbt213292-bib-0181] Orth, J.D. , Conrad, T.M. , Na, J. , Lerman, J.A. , Nam, H. , Feist, A.M. , and Palsson, B.Ø. (2011) A comprehensive genome‐scale reconstruction of *Escherichia coli* metabolism—2011. Mol Syst Biol 7: 535. 21988831 10.1038/msb.2011.65 PMC3261703

[mbt213292-bib-0182] Ouyang, S.P. , Liu, Q. , Fang, L. , and Chen, G.Q. (2007) Construction of *pha*‐operon‐defined knockout mutants of *Pseudomonas putida* KT2442 and their applications in poly(hydroxyalkanoate) production. Macromol Biosci 7: 227–233. 17295412 10.1002/mabi.200600187

[mbt213292-bib-0183] Oztürk, S. , Calik, P. and Ozdamar, T.H. (2016) Fed‐batch biomolecule production by *Bacillus subtilis*: a state of the art review. Trends Biotechnol 34, 329–345. 26775901 10.1016/j.tibtech.2015.12.008

[mbt213292-bib-0184] Palazzotto, E. , and Weber, T. (2018) Omics and multi‐omics approaches to study the biosynthesis of secondary metabolites in microorganisms. Curr Opin Microbiol 45: 109–116. 29656009 10.1016/j.mib.2018.03.004

[mbt213292-bib-0185] Pandit, A.V. , Srinivasan, S. , and Mahadevan, R. (2017) Redesigning metabolism based on orthogonality principles. Nat Commun 8: 1–11. 28555623 10.1038/ncomms15188 PMC5459945

[mbt213292-bib-0186] Park, S.J. , Choi, J.S. , Kim, B.C. , Jho, S.W. , Ryu, J.W. , Park, D. , *et al* (2009) *PutidaNET*: interactome database service and network analysis of *Pseudomonas putida* KT2440. BMC Genom 10: S18. 10.1186/1471-2164-10-S3-S18 PMC2788370 19958481

[mbt213292-bib-0187] Park, M.K. , Lee, S.H. , Yang, K.S. , Jung, S.C. , Lee, J.H. , and Kim, S.C. (2014) Enhancing recombinant protein production with an *Escherichia coli* host strain lacking insertion sequences. Appl Microbiol Biotechnol 98: 6701–6713. 24752842 10.1007/s00253-014-5739-y

[mbt213292-bib-0188] Payne, W.J. , Eagon, R.G. , and Williams, A.K. (1961) Some observations on the physiology of *Pseudomonas natriegens* nov. spec. Antonie van Leeuwenhoek 27: 121–128. 13733692 10.1007/BF02538432

[mbt213292-bib-0189] Peccoud, J. , Blauvelt, M.F. , Cai, Y. , Cooper, K.L. , Crasta, O. , DeLalla, E.C. , *et al* (2008) Targeted development of registries of biological parts. PLoS ONE 3: e2671. 18628824 10.1371/journal.pone.0002671 PMC2441434

[mbt213292-bib-0190] Pérez‐Pantoja, D. , Nikel, P.I. , Chavarría, M. , and de Lorenzo, V. (2013) Endogenous stress caused by faulty oxidation reactions fosters evolution of 2,4‐dinitrotoluene‐degrading bacteria. PLoS Genet 9: e1003764. 24009532 10.1371/journal.pgen.1003764 PMC3757077

[mbt213292-bib-0191] Poblete‐Castro, I. , Becker, J. , Dohnt, K. , Martins dos Santos, V.A.P. , and Wittmann, C. (2012) Industrial biotechnology of *Pseudomonas putida* and related species. Appl Microbiol Biotechnol 93: 2279–2290. 22350258 10.1007/s00253-012-3928-0

[mbt213292-bib-0192] Poblete‐Castro, I. , Borrero de Acuña, J.M. , Nikel, P.I. , Kohlstedt, M. , and Wittmann, C. (2017) Host organism: *Pseudomonas putida* In Industrial Biotechnology: Microorganisms. Wittmann C., and Liao J.C. (eds). Weinheim: Wiley‐VCH Verlag GmbH & Co. KGaA.

[mbt213292-bib-0193] Pontrelli, S. , Chiu, T.Y. , Lan, E.I. , Chen, F.Y. , Chang, P. and Liao, J.C. (2018) *Escherichia coli* as a host for metabolic engineering. Metab Eng, In press. 10.1016/j.ymben.2018.1004.1008 29689382

[mbt213292-bib-0194] Pósfai, G. , Plunkett, G. , Fehér, T. , Frisch, D. , Keil, G.M. , Umenhoffer, K. , *et al* (2006) Emergent properties of reduced‐genome *Escherichia coli* . Science 312: 1044–1046. 16645050 10.1126/science.1126439

[mbt213292-bib-0195] Price, M.N. , Wetmore, K.M. , Waters, R.J. , Callaghan, M. , Ray, J. , Liu, H. , *et al* (2018) Mutant phenotypes for thousands of bacterial genes of unknown function. Nature 557: 503–509. 29769716 10.1038/s41586-018-0124-0

[mbt213292-bib-0196] Prieto, A. , Escapa, I.F. , Martínez, V. , Dinjaski, N. , Herencias, C. , de la Peña, F. , *et al* (2016) A holistic view of polyhydroxyalkanoate metabolism in *Pseudomonas putida* . Environ Microbiol 18: 341–357. 25556983 10.1111/1462-2920.12760

[mbt213292-bib-0197] Puchałka, J. , Oberhardt, M.A. , Godinho, M. , Bielecka, A. , Regenhardt, D. , Timmis, K.N. , *et al* (2008) Genome‐scale reconstruction and analysis of the *Pseudomonas putida* KT2440 metabolic network facilitates applications in biotechnology. PLoS Comput Biol 4, e1000210. 18974823 10.1371/journal.pcbi.1000210 PMC2563689

[mbt213292-bib-0198] Qi, L.S. , Larson, M.H. , Gilbert, L.A. , Doudna, J.A. , Weissman, J.S. , Arkin, A.P. , and Lim, W.A. (2013) Repurposing CRISPR as an RNA‐guided platform for sequence‐specific control of gene expression. Cell 152: 1173–1183. 23452860 10.1016/j.cell.2013.02.022 PMC3664290

[mbt213292-bib-0199] Ragauskas, A.J. , Beckham, G.T. , Biddy, M.J. , Chandra, R. , Chen, F. , Davis, M.F. , *et al* (2014) Lignin valorization: improving lignin processing in the biorefinery. Science 344: 1246843. 24833396 10.1126/science.1246843

[mbt213292-bib-0200] Ramos, J.L. , Duque, E. , Gallegos, M.T. , Godoy, P. , Ramos‐González, M.I. , Rojas, A. , *et al* (2002) Mechanisms of solvent tolerance in Gram‐negative bacteria. Annu Rev Microbiol 56: 743–768. 12142492 10.1146/annurev.micro.56.012302.161038

[mbt213292-bib-0201] Ravi, K. , García‐Hidalgo, J. , Gorwa‐Grauslund, M.F. , and Lidén, G. (2017) Conversion of lignin model compounds by *Pseudomonas putida* KT2440 and isolates from compost. Appl Microbiol Biotechnol 101: 5059–5070. 28299400 10.1007/s00253-017-8211-y PMC5486835

[mbt213292-bib-0202] Riggs, A.D. (1981) Bacterial production of human insulin. Diabetes Care 4: 64–68. 7009117 10.2337/diacare.4.1.64

[mbt213292-bib-0203] Røkke, G. , Korvald, E. , Pahr, J. , Oyås, O. , and Lale, R. (2014) *BioBrick* assembly standards and techniques and associated software tools. Methods Mol Biol 1116: 1–24. 24395353 10.1007/978-1-62703-764-8_1

[mbt213292-bib-0204] Rosano, G.L. , and Ceccarelli, E.A. (2014) Recombinant protein expression in *Escherichia coli*: advances and challenges. Front Microbiol 5: 172. 24860555 10.3389/fmicb.2014.00172 PMC4029002

[mbt213292-bib-0205] Röttig, A. , Hauschild, P. , Madkour, M.H. , Al‐Ansari, A.M. , Almakishah, N.H. , and Steinbüchel, A. (2016) Analysis and optimization of triacylglycerol synthesis in novel oleaginous *Rhodococcus* and *Streptomyces* strains isolated from desert soil. J Biotechnol 225: 48–56. 27034020 10.1016/j.jbiotec.2016.03.040

[mbt213292-bib-0206] Ruffing, A.M. , Jensen, T.J. and Strickland, L.M. (2016) Genetic tools for advancement of *Synechococcus* sp. PCC 7002 as a cyanobacterial *chassis* . Microb Cell Fact 15, 1–14. 27832791 10.1186/s12934-016-0584-6 PMC5105302

[mbt213292-bib-0207] Rugbjerg, P. , Myling‐Petersen, N. , Porse, A. , Sarup‐Lytzen, K. , and Sommer, M.O.A. (2018) Diverse genetic error modes constrain large‐scale bio‐based production. Nat Commun 9: 787. 29463788 10.1038/s41467-018-03232-w PMC5820350

[mbt213292-bib-0208] Rujananon, R. , Prasertsan, P. , and Phongdara, A. (2014) Biosynthesis of 1,3‐propanediol from recombinant *E*. *coli* by optimization process using pure and crude glycerol as a sole carbon source under two‐phase fermentation system. World J Microbiol Biotechnol 30: 1359–1368. 24249578 10.1007/s11274-013-1556-1

[mbt213292-bib-0209] Sabra, W. , Groeger, C. , and Zeng, A.P. (2016) Microbial cell factories for diol production. Adv Biochem Eng Biotechnol 155: 165–197. 26475465 10.1007/10_2015_330

[mbt213292-bib-0210] Salgado, J.M. , Rodríguez‐Solana, R. , Curiel, J.A. , de las Rivas, B. , Muñoz, R. and Domínguez, J.M. (2014) Bioproduction of 4‐vinylphenol from corn cob alkaline hydrolyzate in two‐phase extractive fermentation using free or immobilized recombinant *E*. *coli* expressing *pad* gene. Enzyme Microb Technol 58‐59: 2, 2–28. 10.1016/j.enzmictec.2014.02.005 24731821

[mbt213292-bib-0211] Samantaray, S. and Mallick, N. (2012) Production and characterization of poly‐β‐hydroxybutyrate (PHB) polymer from *Aulosira fertilissima* . J Appl Phycol 24, 803–814.

[mbt213292-bib-0212] Sánchez‐Pascuala, A. , de Lorenzo, V. , and Nikel, P.I. (2017) Refactoring the Embden‐Meyerhof‐Parnas pathway as a whole of portable *GlucoBricks* for implantation of glycolytic modules in Gram‐negative bacteria. ACS Synth Biol 6: 793–805. 28121421 10.1021/acssynbio.6b00230 PMC5440799

[mbt213292-bib-0213] Santos, P.M. , Benndorf, D. , and Sá‐Correia, I. (2004) Insights into *Pseudomonas putida* KT2440 response to phenol‐induced stress by quantitative proteomics. Proteomics 4: 2640–2652. 15352239 10.1002/pmic.200300793

[mbt213292-bib-0214] Sarria, S. , Kruyer, N.S. , and Peralta‐Yahya, P. (2017) Microbial synthesis of medium‐chain chemicals from renewables. Nat Biotechnol 35: 1158–1166. 29220020 10.1038/nbt.4022

[mbt213292-bib-0215] Savakis, P. , and Hellingwerf, K.J. (2015) Engineering cyanobacteria for direct biofuel production from CO_2_ . Curr Opin Biotechnol 33: 8–14. 25305544 10.1016/j.copbio.2014.09.007

[mbt213292-bib-0216] Schallmey, M. , Singh, A. , and Ward, O.P. (2004) Developments in the use of *Bacillus* species for industrial production. Can J Microbiol 50: 1–17. 15052317 10.1139/w03-076

[mbt213292-bib-0217] Schmitz, S. , Nies, S. , Wierckx, N. , Blank, L.M. , and Rosenbaum, M.A. (2015) Engineering mediator‐based electroactivity in the obligate aerobic bacterium *Pseudomonas putida* KT2440. Front Microbiol 6: 284. 25914687 10.3389/fmicb.2015.00284 PMC4392322

[mbt213292-bib-0218] Segall‐Shapiro, T.H. , Sontag, E.D. , and Voigt, C.A. (2018) Engineered promoters enable constant gene expression at any copy number in bacteria. Nat Biotechnol 36: 352–358. 29553576 10.1038/nbt.4111

[mbt213292-bib-0219] Sengupta, S. , Jonnalagadda, S. , Goonewardena, L. , and Juturu, V. (2015) Metabolic engineering of a novel muconic acid biosynthesis pathway *via* 4‐hydroxybenzoic acid in *Escherichia coli* . Appl Environ Microbiol 81: 8037–8043. 26362984 10.1128/AEM.01386-15 PMC4651072

[mbt213292-bib-0220] Shen, C.R. , Lan, E.I. , Dekishima, Y. , Baez, A. , Cho, K.M. , and Liao, J.C. (2011) Driving forces enable high‐titer anaerobic 1‐butanol synthesis in *Escherichia coli* . Appl Environ Microbiol 77: 2905–2915. 21398484 10.1128/AEM.03034-10 PMC3126405

[mbt213292-bib-0221] Shepelin, D. , Hansen, A.S.L. , Lennen, R.M. , Luo, H. , and Herrgård, M.J. (2018) Selecting the best: evolutionary engineering of chemical production in microbes. Genes 9: 249. 10.3390/genes9050249 PMC5977189 29751691

[mbt213292-bib-0222] Shi, T. , Wang, Y. , Wang, Z. , Wang, G. , Liu, D. , Fu, J. , *et al* (2014) Deregulation of purine pathway in *Bacillus subtilis* and its use in riboflavin biosynthesis. Microb Cell Fact 13: 101. 25023436 10.1186/s12934-014-0101-8 PMC4223553

[mbt213292-bib-0223] Shih, I.L. , and Van, Y.T. (2001) The production of poly(γ‐glutamic acid) from microorganisms and its various applications. Bioresour Technol 79: 207–225. 11499575 10.1016/s0960-8524(01)00074-8

[mbt213292-bib-0224] Shiloach, J. , and Fass, R. (2005) Growing *E*. *coli* to high cell density—A historical perspective on method development. Biotechnol Adv 23: 345–357. 15899573 10.1016/j.biotechadv.2005.04.004

[mbt213292-bib-0225] Silva‐Rocha, R. , Martínez‐García, E. , Calles, B. , Chavarría, M. , Arce‐Rodríguez, A. , de Las Heras, A. , *et al* (2013) The Standard European Vector Architecture (*SEVA*): a coherent platform for the analysis and deployment of complex prokaryotic phenotypes. Nucleic Acids Res 41: D666–D675. 23180763 10.1093/nar/gks1119 PMC3531073

[mbt213292-bib-0226] Singh, A.K. , and Mallick, N. (2018) Advances in cyanobacterial polyhydroxyalkanoates production. FEMS Microbiol Lett 364: 1–13. 10.1093/femsle/fnx189 28961962

[mbt213292-bib-0227] Singh, R. , Kumar, M. , Mittal, A. , and Kumar, P. (2016) Microbial enzymes: industrial progress in 21st century. *3* . Biotech 6: 1–15. 10.1007/s13205-016-0485-8 PMC4991975 28330246

[mbt213292-bib-0228] Smanski, M.J. , Zhou, H. , Claesen, J. , Shen, B. , Fischbach, M.A. , and Voigt, C.A. (2016) Synthetic biology to access and expand nature's chemical diversity. Nat Rev Microbiol 14: 135–149. 26876034 10.1038/nrmicro.2015.24 PMC5048682

[mbt213292-bib-0229] Sohn, S.B. , Kim, T.Y. , Park, J.M. , and Lee, S.Y. (2010) *In silico* genome‐scale metabolic analysis of *Pseudomonas putida* KT2440 for polyhydroxyalkanoate synthesis, degradation of aromatics and anaerobic survival. Biotechnol J 5: 739–750. 20540110 10.1002/biot.201000124

[mbt213292-bib-0230] Song, Y. , Nikoloff, J.M. , and Zhang, D. (2015) Improving protein production on the level of regulation of both expression and secretion pathways in *Bacillus subtilis* . J Microbiol Biotechnol 25: 963–977. 25737123 10.4014/jmb.1501.01028

[mbt213292-bib-0231] Spasic, J. , Mandic, M. , Djokic, L. , and Nikodinovic‐Runic, J. (2018) *Streptomyces* spp. in the biocatalysis toolbox. Appl Microbiol Biotechnol 102: 3513–3536. 29502181 10.1007/s00253-018-8884-x

[mbt213292-bib-0232] Spizizen, J. (1958) Transformation of biochemically deficient strains of *Bacillus subtilis* by deoxyribonucleate. Proc Natl Acad Sci USA 44: 1072–1078. 16590310 10.1073/pnas.44.10.1072 PMC528696

[mbt213292-bib-0233] Stahmann, K.P. , Revuelta, J.L. , and Seulberger, H. (2000) Three biotechnical processes using *Ashbya gossypii*,* Candida famata*, or *Bacillus subtilis* compete with chemical riboflavin production. Appl Microbiol Biotechnol 53: 509–516. 10855708 10.1007/s002530051649

[mbt213292-bib-0234] Steinbüchel, A. , and Lütke‐Eversloh, T. (2003) Metabolic engineering and pathway construction for biotechnological production of relevant polyhydroxyalkanoates in microorganisms. Biochem Eng J 16: 81–96.

[mbt213292-bib-0235] Stephanopoulos, G. (2012) Synthetic biology and metabolic engineering. ACS Synth Biol 1: 514–525. 23656228 10.1021/sb300094q

[mbt213292-bib-0236] Sun, J. , Yu, H. , Chen, J. , Luo, H. , and Shen, Z. (2016) Ammonium acrylate biomanufacturing by an engineered *Rhodococcus ruber* with nitrilase overexpression and double‐knockout of nitrile hydratase and amidase. J Ind Microbiol Biotechnol 43: 1631–1639. 27761748 10.1007/s10295-016-1840-9

[mbt213292-bib-0237] Tan, D. , Xue, Y.S. , Aibaidula, G. , and Chen, G.Q. (2011) Unsterile and continuous production of polyhydroxybutyrate by *Halomonas* TD01. Bioresour Technol 102: 8130–8136. 21680179 10.1016/j.biortech.2011.05.068

[mbt213292-bib-0238] Tanaka, T. , Fujita, K.I. , Takenishi, S. , and Taniguchi, M. (1997) Existence of an optically heterogeneous peptide unit in poly(γ‐glutamic acid) produced by *Bacillus subtilis* . J Ferment Bioeng 84: 361–364.

[mbt213292-bib-0239] Tang, K. , Yang, Y. , Lin, D. , Li, S. , Zhou, W. , Han, Y. , *et al* (2016) Genomic, physiologic, and proteomic insights into metabolic versatility in *Roseobacter* clade bacteria isolated from deep‐sea water. Sci Rep 6: 35528. 27762339 10.1038/srep35528 PMC5071866

[mbt213292-bib-0240] Tao, W. , Lv, L. , and Chen, G.Q. (2017) Engineering *Halomonas* species TD01 for enhanced polyhydroxyalkanoates synthesis *via* CRISPRi. Microb Cell Fact 16: 48. 28381263 10.1186/s12934-017-0655-3 PMC5382479

[mbt213292-bib-0241] Terakawa, A. , Natsume, A. , Okada, A. , Nishihata, S. , Kuse, J. , Tanaka, K. , *et al* (2016) *Bacillus subtilis* 5'‐nucleotidases with various functions and substrate specificities. BMC Microbiol 16: 249. 27784292 10.1186/s12866-016-0866-5 PMC5080769

[mbt213292-bib-0242] Terpe, K. (2006) Overview of bacterial expression systems for heterologous protein production: from molecular and biochemical fundamentals to commercial systems. Appl Microbiol Biotechnol 72: 211–222. 16791589 10.1007/s00253-006-0465-8

[mbt213292-bib-0243] Tiso, T. , Wierckx, N. , and Blank, L.M. (2014) Non‐pathogenic *Pseudomonas* as platform for industrial biocatalysis In Industrial Biocatalysis. Grunwald P. (ed). Boca Raton, FL: CRC Press, pp. 323–372.

[mbt213292-bib-0244] Tiso, T. , Zauter, R. , Tulke, H. , Leuchtle, B. , Li, W.J. , Behrens, B. , *et al* (2017) Designer rhamnolipids by reduction of congener diversity: production and characterization. Microb Cell Fact 16: 225. 29241456 10.1186/s12934-017-0838-y PMC5729600

[mbt213292-bib-0245] Tjalsma, H. , Antelmann, H. , Jongbloed, J.D.H. , Braun, P.G. , Darmon, E. , Dorenbos, R. , *et al* (2004) Proteomics of protein secretion by *Bacillus subtilis*: separating the “secrets” of the secretome. Microbiol Mol Biol Rev 68: 207–233. 15187182 10.1128/MMBR.68.2.207-233.2004 PMC419921

[mbt213292-bib-0246] Tokuyama, K. , Toya, Y. , Horinouchi, T. , Furusawa, C. , Matsuda, F. and Shimizu, H. (2018) Application of adaptive laboratory evolution to overcome a flux limitation in an *Escherichia coli* production strain. Biotechnol Bioeng, 115, 1542–1551. 29457640 10.1002/bit.26568

[mbt213292-bib-0247] Unthan, S. , Baumgart, M. , Radek, A. , Herbst, M. , Siebert, D. , Brühl, N. , *et al* (2015) *Chassis* organism from *Corynebacterium glutamicum*—A top‐down approach to identify and delete irrelevant gene clusters. Biotechnol J 10: 290–301. 25139579 10.1002/biot.201400041 PMC4361050

[mbt213292-bib-0248] Verhoef, S. , Wierckx, N. , Westerhof, R.G.M. , de Winde, J.H. , and Ruijssenaars, H.J. (2009) Bioproduction of *p*‐hydroxystyrene from glucose by the solvent‐tolerant bacterium *Pseudomonas putida* S12 in a two‐phase water‐decanol fermentation. Appl Environ Microbiol 75: 931–936. 19060171 10.1128/AEM.02186-08 PMC2643573

[mbt213292-bib-0249] Wang, H.H. , Isaacs, F.J. , Carr, P.A. , Sun, Z.Z. , Xu, G. , Forest, C.R. , and Church, G.M. (2009) Programming cells by multiplex genome engineering and accelerated evolution. Nature 460: 894–898. 19633652 10.1038/nature08187 PMC4590770

[mbt213292-bib-0250] Wang, B. , Wang, J. , Zhang, W. , Meldrum, D.R. , and Peebles, C.A.M. (2012) Application of synthetic biology in cyanobacteria and algae. Front Microbiol 3: 1–15. 23049529 10.3389/fmicb.2012.00344 PMC3446811

[mbt213292-bib-0251] Wang, B. , Pugh, S. , Nielsen, D.R. , Zhang, W. , and Meldrum, D.R. (2013) Engineering cyanobacteria for photosynthetic production of 3‐hydroxybutyrate directly from CO_2_ . Metab Eng 16: 68–77. 23333586 10.1016/j.ymben.2013.01.001

[mbt213292-bib-0252] Wang, J. , Niyompanich, S. , Tai, Y.‐S. , Wang, J. , Bai, W. , Mahida, P. , *et al* (2016a) Engineering highly efficient *E*. *coli* strain for mevalonate fermentation by chromosomal integration. Appl Environ Microbiol 82: 7176–7184. 27736790 10.1128/AEM.02178-16 PMC5118928

[mbt213292-bib-0253] Wang, R. , Gallant, É. and Seyedsayamdost, R. (2016b) Investigation of the genetics and biochemistry of roseobacticide production in the *Roseobacter* clade bacterium *Phaeobacter inhibens* . mBio 7, e02118‐15. 27006458 10.1128/mBio.02118-15 PMC4807370

[mbt213292-bib-0254] Weaver, L.M. , and Herrmann, K.M. (1990) Cloning of an *aroF* allele encoding a tyrosine‐insensitive 3‐deoxy‐D‐arabino‐heptulosonate 7‐phosphate synthase. J Bacteriol 172: 6581–6584. 1977738 10.1128/jb.172.11.6581-6584.1990 PMC526849

[mbt213292-bib-0255] Weinstock, M.T. , Hesek, E.D. , Wilson, C.M. , and Gibson, D.G. (2016) *Vibrio natriegens* as a fast‐growing host for molecular biology. Nat Methods 13: 849–851. 27571549 10.1038/nmeth.3970

[mbt213292-bib-0256] Wendisch, V.F. (2014) Microbial production of amino acids and derived chemicals: synthetic biology approaches to strain development. Curr Opin Biotechnol 30: 51–58. 24922334 10.1016/j.copbio.2014.05.004

[mbt213292-bib-0257] Wendisch, V.F. , Brito, L.F. , Gil‐López, M. , Hennig, G. , Pfeifenschneider, J. , Sgobba, E. , and Veldmann, K.H. (2016) The flexible feedstock concept in Industrial Biotechnology: metabolic engineering of *Escherichia coli*,* Corynebacterium glutamicum*,* Pseudomonas*,* Bacillus* and yeast strains for access to alternative carbon sources. J Biotechnol 234: 139–157. 27491712 10.1016/j.jbiotec.2016.07.022

[mbt213292-bib-0258] Wendt, K.E. , Ungerer, J. , Cobb, R.E. , Zhao, H. , and Pakrasi, H.B. (2016) CRISPR/Cas9 mediated targeted mutagenesis of the fast growing cyanobacterium *Synechococcus elongatus* UTEX 2973. Microb Cell Fact 15: 115. 27339038 10.1186/s12934-016-0514-7 PMC4917971

[mbt213292-bib-0259] Westers, L. , Westers, H. , and Quax, W.J. (2004) *Bacillus subtilis* as cell factory for pharmaceutical proteins: a biotechnological approach to optimize the host organism. Biochim Biophys Acta 1694: 299–310. 15546673 10.1016/j.bbamcr.2004.02.011

[mbt213292-bib-0260] Wierckx, N.J.P. , Ballerstedt, H. , de Bont, J.M. , and Wery, J. (2005) Engineering of solvent‐tolerant *Pseudomonas putida* S12 for bioproduction of phenol from glucose. Appl Environ Microbiol 71: 8221–8227. 16332806 10.1128/AEM.71.12.8221-8227.2005 PMC1317433

[mbt213292-bib-0261] Winsor, G.L. , Griffiths, E.J. , Lo, R. , Dhillon, B.K. , Shay, J.A. , and Brinkman, F.S.L. (2016) Enhanced annotations and features for comparing thousands of *Pseudomonas* genomes in the *Pseudomonas* genome database. Nucleic Acids Res 44: D646–D653. 26578582 10.1093/nar/gkv1227 PMC4702867

[mbt213292-bib-0262] Wittgens, A. , Tiso, T. , Arndt, T.T. , Wenk, P. , Hemmerich, J. , Müller, C. , *et al* (2011) Growth independent rhamnolipid production from glucose using the non‐pathogenic *Pseudomonas putida* KT2440. Microb Cell Fact 10: 80. 21999513 10.1186/1475-2859-10-80 PMC3258213

[mbt213292-bib-0263] Wu, X.C. , Lee, W. , Tran, L. , and Wong, S.L. (1991) Engineering a *Bacillus subtilis* expression‐secretion system with a strain deficient in six extracellular proteases. J Bacteriol 173: 4952–4958. 1907264 10.1128/jb.173.16.4952-4958.1991 PMC208183

[mbt213292-bib-0264] Wu, S.C. , Yeung, J.C. , Duan, Y. , Ye, R. , Szarka, S.J. , Habibi, H.R. , and Wong, S.L. (2002) Functional production and characterization of a fibrin‐specific single‐chain antibody fragment from *Bacillus subtilis*: effects of molecular chaperones and a wall‐bound protease on antibody fragment production. Appl Environ Microbiol 68: 3261–3269. 12089002 10.1128/AEM.68.7.3261-3269.2002 PMC126797

[mbt213292-bib-0265] Wynands, B. , Lenzen, C. , Otto, M. , Koch, F. , Blank, L.M. , and Wierckx, N. (2018) Metabolic engineering of *Pseudomonas taiwanensis* VLB120 with minimal genomic modifications for high‐yield phenol production. Metab Eng 47: 121–133. 29548982 10.1016/j.ymben.2018.03.011

[mbt213292-bib-0266] Xavier, J.C. , Patil, K.R. , and Rocha, I. (2014) Systems biology perspectives on minimal and simpler cells. Microbiol Mol Biol Rev 78: 487–509. 25184563 10.1128/MMBR.00050-13 PMC4187685

[mbt213292-bib-0267] Yim, H. , Haselbeck, R. , Niu, W. , Pujol‐Baxley, C. , Burgard, A. , Boldt, J. , *et al* (2011) Metabolic engineering of *Escherichia coli* for direct production of 1,4‐butanediol. Nat Chem Biol 7: 445–452. 21602812 10.1038/nchembio.580

[mbt213292-bib-0268] Yin, J. , Fu, X.Z. , Wu, Q. , Chen, J.C. , and Chen, G.Q. (2014) Development of an enhanced chromosomal expression system based on porin synthesis operon for halophile *Halomonas* sp. Appl Microbiol Biotechnol 98: 8987–8997. 25070598 10.1007/s00253-014-5959-1

[mbt213292-bib-0269] Yishai, O. , Goldbach, L. , Tenenboim, H. , Lindner, S.N. , and Bar‐Even, A. (2017) Engineered assimilation of exogenous and endogenous formate in *Escherichia coli* . ACS Synth Biol 6: 1722–1731. 28558223 10.1021/acssynbio.7b00086

[mbt213292-bib-0270] Yu, Y. , You, L. , Liu, D. , Hollinshead, W. , Tang, Y.J. , and Zhang, F. (2013) Development of *Synechocystis* sp. PCC6803 as a phototrophic cell factory. Marine Drugs 11: 2894–2916. 23945601 10.3390/md11082894 PMC3766872

[mbt213292-bib-0271] Yu, S. , Plan, M.R. , Winter, G. , and Krömer, J.O. (2016) Metabolic engineering of *Pseudomonas putida* KT2440 for the production of *para*‐hydroxybenzoic acid. Front Bioeng Biotechnol 4: 90. 27965953 10.3389/fbioe.2016.00090 PMC5124731

[mbt213292-bib-0272] Yus, E. , Maier, T. , Michalodimitrakis, K. , van Noort, V. , Yamada, T. , Chen, W.H. , *et al* (2009) Impact of genome reduction on bacterial metabolism and its regulation. Science 326: 1263–1268. 19965476 10.1126/science.1177263

[mbt213292-bib-0273] Zeng, W. , Chen, G. , Wu, H. , Wang, J. , Liu, Y. , Guo, Y. , and Liang, Z. (2016) Improvement of *Bacillus subtilis* for poly‐γ‐glutamic acid production by genome shuffling. Microb Biotechnol 9: 824–833. 27562078 10.1111/1751-7915.12405 PMC5072198

[mbt213292-bib-0274] Zhang, F. , and Keasling, J. (2011) Biosensors and their applications in microbial metabolic engineering. Trends Microbiol 19: 323–329. 21664818 10.1016/j.tim.2011.05.003

[mbt213292-bib-0275] Zhao, H. , Zhang, H.M. , Chen, G.Q. , Chen, X. , Li, T. , Wu, Q. , and Ouyang, Q. (2017) Novel T*7*‐like expression systems used for *Halomonas* . Metab Eng 39: 128–140. 27889295 10.1016/j.ymben.2016.11.007

[mbt213292-bib-0276] Zhou, Y.J. , Gao, W. , Rong, Q. , Jin, G. , Chu, H. , Liu, W. , *et al* (2012) Modular pathway engineering of diterpenoid synthases and the mevalonic acid pathway for miltiradiene production. J Am Chem Soc 134: 3234–3241. 22280121 10.1021/ja2114486

[mbt213292-bib-0277] Zobel, S. , Benedetti, I. , Eisenbach, L. , de Lorenzo, V. , Wierckx, N. , and Blank, L.M. (2015) Tn*7*‐Based device for calibrated heterologous gene expression in *Pseudomonas putida* . ACS Synth Biol 4: 1341–1351. 26133359 10.1021/acssynbio.5b00058

